# S-Nitrosylation in Cardiovascular Disorders: The State of the Art

**DOI:** 10.3390/biom15081073

**Published:** 2025-07-24

**Authors:** Caiyun Mao, Jieyou Zhao, Nana Cheng, Zihang Xu, Haoming Ma, Yunjia Song, Xutao Sun

**Affiliations:** 1 Department of Pharmacology, School of Basic Medical Sciences, Heilongjiang University of Chinese Medicine, Harbin 150040, China; 2 Department of Typhoid, School of Basic Medical Sciences, Heilongjiang University of Chinese Medicine, Harbin 150040, China; 3 Department of Chinese Internal Medicine, First Affiliated Hospital, Heilongjiang University of Chinese Medicine, Harbin 150040, China

**Keywords:** S-nitrosylation, hypertension, pulmonary arterial hypertension, atherosclerosis, heart failure, myocardial infarction, arrhythmia

## Abstract

Protein S-nitrosylation is a selective post-translational modification in which a nitrosyl group is covalently attached to the reactive thiol group of cysteine, forming S-nitrosothiol. This modification plays a pivotal role in modulating physiological and pathological cardiovascular processes by altering protein conformation, activity, stability, and other post-translational modifications. It is instrumental in regulating vascular and myocardial systolic and diastolic functions, vascular endothelial cell and cardiomyocyte apoptosis, and cardiac action potential and repolarization. Aberrant S-nitrosylation levels are implicated in the pathogenesis of various cardiovascular diseases, including systemic hypertension, pulmonary arterial hypertension, atherosclerosis, heart failure, myocardial infarction, arrhythmia, and diabetic cardiomyopathy. Insufficient S-nitrosylation leads to impaired vasodilation and increased vascular resistance, while excessive S-nitrosylation contributes to cardiac hypertrophy and myocardial fibrosis, thereby accelerating ventricular remodeling. This paper reviews the S-nitrosylated proteins in the above-mentioned diseases and their impact on these conditions through various signaling pathways, with the aim of providing a theoretical foundation for the development of novel therapeutic strategies or drugs targeting S-nitrosylated proteins.

## 1. Introduction

**Figure 1:**
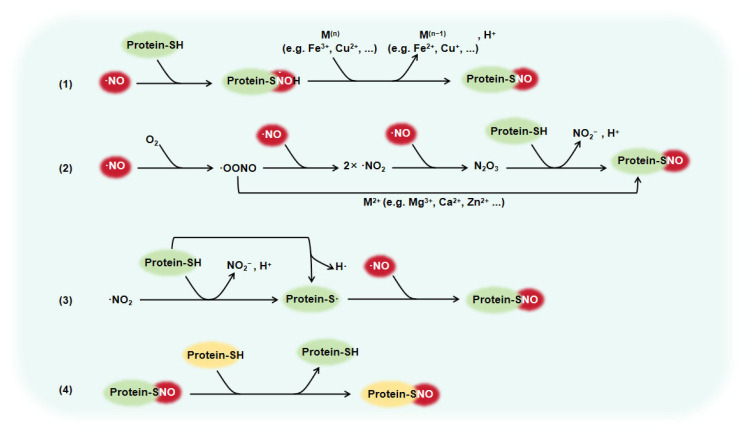
Formation pathway of S-nitrosothiol in mammals. (1) Direct addition catalyzed by transition metal; (2) Autoxidation of NO; (3) Free radical recombination; (4) Transnitrosation. “M” indicates a metal or other cation. Transition metals, such as copper (II) or iron (III), act as electron acceptors. Divalent cations, like zinc, act as catalysts.

The concept of S-nitrosylation was first proposed by Stamler et al. in 1992 to describe the reaction of thiols exposed to nitric oxide (NO) [1]. NO is a free radical containing a single unpaired electron. This characteristic renders NO highly reactive and short-lived, with a biological half-life of just a few seconds or less [2]. The characteristic also renders protein S-nitrosylation a reversible post-translational modification that involves attaching a nitrosyl group to the active thiol group of cysteine to form S-nitrosothiol [3]. This reversibility is biologically significant, as an S-nitrosylation product can act as a feedback mechanism that regulates NO synthesis, thereby contributing to redox homeostasis and signal modulation. In mammals, S-nitrosothiol formation generally occurs through the following pathways [4] (Figure 1): (1) Direct addition catalyzed by a transition metal: NO reacts directly with a protein thiol at a metal-containing enzyme active site to form S-nitrosothiol. Transition metal ions such as iron (III) and copper (II) contribute to the activation of NO molecules or the oxidation of thiol groups, thereby enhancing the reaction efficiency. (2) Autoxidation of NO: Under the presence of oxygen, NO can spontaneously oxidize to form N_2_O_3_, which is a potent nitrosating agent capable of reacting with thiol groups to produce S-nitrosothiols. (3) Free radical recombination: NO, in the form of •NO_2_ radicals, undergoes a recombination reaction with thiyl radicals to form S-nitrosothiols. (4) Transnitrosation: The formed protein S-nitrosothiols can be transferred to other free thiols through transnitrosation reactions. This reaction can diffuse and transmit NO-mediated signaling, affecting the functions of multiple proteins. Previous studies have shown that protein S-nitrosylation has a cardioprotective effect, and that the dysregulation of S-nitrosylation levels is associated with the pathophysiological mechanisms of cardiovascular diseases such as hypertension, pulmonary arterial hypertension (PAH), ischemic heart disease, and heart failure [5,6]. Therefore, S-nitrosylation is a potential target for cardiovascular protection.

Endogenous NO is predominantly synthesized from L-arginine by three nitric oxide synthases (NOSs): neuronal NOS (nNOS), inducible NOS (iNOS), and endothelial NOS (eNOS) [7]. NOS activity is oxygen-dependent, and impaired function under hypoxic conditions reduces NO production [8]. During such instances, nitrate and nitrite in the body serve as “reserve resources,” being converted to NO through alternative pathways [9]. Nitrite, in particular, can act as a regulator, regulating cardiac function by promoting the S-nitrosylation of proteins. This paper explores the roles of NOS and nitrate/nitrite-mediated protein S-nitrosylation in heart diseases, including systemic hypertension, PAH, atherosclerosis, heart failure, myocardial infarction, arrhythmia, and diabetic cardiomyopathy, and examines the potential of S-nitrosylation regulation as a novel therapeutic strategy, offering new insights into the treatment of heart diseases and the development of drugs targeting S-nitrosylation.

## 2. The Role of Protein S-Nitrosylation in Vascular Diseases

### 2.1. Effect of Protein S-Nitrosylation in Systemic Hypertension

**Figure 2:**
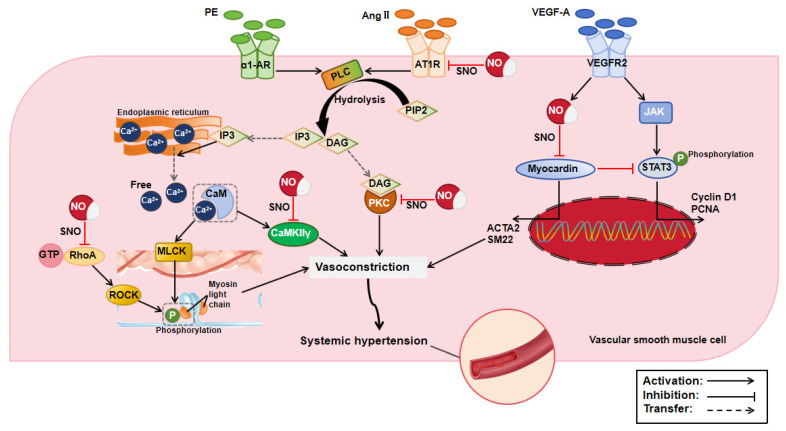
Mechanism of protein S-nitrosylation in systemic hypertension. ACTA2: actin alpha 2, AngII: angiotensin II, AT1R: angiotensin type 1 receptor, CaMKIIγ: calcium/calmodulin-dependent protein kinase II γ, CaM: calmodulin, DAG: diacylglycerol, GTP: guanosine triphosphate, IP3: inositol triphosphate, JAK: Janus kinase, MLCK: myosin light-chain kinase, NO: nitric oxide, PCNA: proliferating cell nuclear antigen, PE: phenylephrine, PIP2: phosphatidylinositol 4, 5-diphosphate, PKC: protein kinase C, PLC: phospholipase C, RhoA: Ras homolog gene family member A, ROCK: Rho-associated kinase, SNO: S-nitrosylation, STAT3: signal transducer and activator of transcription 3, VEGF-A: vascular endothelial growth factor-A, VEGFR2: vascular endothelial growth factor receptor 2, α1-AR: α1-adrenergic receptor.

Hypertension is a complex pathophysiological state characterized by sustained blood pressure elevation, which leads to vasoconstriction and vascular dysfunction. Numerous studies have now shown that NO-mediated S-nitrosylation can modify a variety of proteins (Table 1), thereby affecting the phenotype of vascular smooth muscle cells (VSMCs), the responsiveness of VSMCs to vasoconstrictor substances, and the activation of the RhoA/Rho kinase signaling pathway, thus alleviating the pathophysiological processes of hypertension (Figure 2). In general, VSMCs primarily exhibit contractile phenotypes that maintain the vascular contraction function, while myocardin is an important transcriptional cofactor that can regulate the VSMCs’ phenotype. However, S-nitrosylation of myocardin attenuates its promoting effect on the expression of contractile phenotypic markers and its inhibiting effect on STAT3 activation, which subsequently results in VSMC proliferation [10]. The transition of VSMCs from a contractile phenotype to a proliferative phenotype alleviates vascular over-contraction under the condition of hypertension. Mechanistically, the persistent contractile state of VSMCs is attributed to the increased levels of angiotensin II (AngII) and endothelin-1 (ET-1) as well as the over-activity of the sympathetic nervous system that accompany hypertension. An oral nitrite treatment can reduce the sensitivity of VSMCs to the vasoconstrictor substance AngII. An increased S-nitrosylation of protein kinase C (PKC) attenuates the contractile response of the aorta to AngII [11]. Meanwhile, S-nitrosylation of AT1R destroys its binding affinity to AngII, which inhibits Ca^2+^ mobilization and subsequent aortic contractions under AngII stimulation [11,12]. The reduction in intracellular Ca^2+^ concentration is of significance for alleviating the contraction of the aorta under the condition of hypertension. S-nitrosylation can directly limit Ca^2+^ influx by reducing the total and surface protein levels of Cav1.2 channels under hypertension [13] and modulating the function of STIM2 [14]. The release of NO donors was also positively correlated with the degree of suppression of ET-1 secretion. In cultured porcine aortic endothelial cells, the NOS inhibitor L-NNA increased phorbol ester-stimulated ET-1 secretion, whereas the selective soluble guanylyl cyclase (sGC) inhibitor ODQ had no effect at basal and stimulated levels [15]. This suggests that S-nitrosylation may be responsible for the NO-induced reduction in ET-1 secretion in endothelial cells instead of cGMP, but it remains undefined.

Sympathetic hyperactivity increases the release of adrenaline, leading to the abnormal activation of adrenergic receptors (ARs) and sustained VSMC contractions. The oral administration of sodium nitrite prevents increased α1-adrenergic vasoconstriction in phenylephrine-induced hypertensive rats. This effect is associated with increased S-nitrosylation of calcium/calmodulin-dependent protein kinase II γ (CaMKIIγ) in the rat aorta. CaMKIIγ is a multifunctional protein that mediates α1-AR signaling. S-nitrosylation decreases its activity, thereby inhibiting vasoconstriction [16]. Meanwhile, sympathetic hyperactivity alters cell surface β-ARs, activating multiple intracellular signaling pathways. These pathways are inhibited by the S-nitrosylation of β-arrestin 2, which regulates most of G protein-coupled receptor (GPCR) signaling. S-nitrosylation alters the structure and function of β-arrestin 2 and promotes its binding to a clathrin heavy chain and β-adaptin, thereby accelerating β-AR internalization [17]. This effect not only reduces the number of receptors on the cell surface and sensitivity to adrenaline but may also affect the recycling and degradation of receptors. By this mechanism, S-nitrosylation of β-arrestin 2 may help to inhibit the development and maintenance of hypertension. Furthermore, mechanical stress in the hypertensive environment activates the RhoA/Rho kinase pathway, which promotes VSMC contraction by increasing the phosphorylation of myosin light chains. S-nitrosylation of the RhoA inhibits RhoA/Rho kinase signaling, thereby inhibiting myosin light chain phosphorylation and vasoconstriction [18]. S-nitrosylation sometimes plays a negative role in hypertension. For example, in a transverse aortic constriction (TAC)-induced acute hypertension rat model, iNOS activation increased the overall level of protein S-nitrosylation in adventitial fibroblasts, promoting an excessive deposition of collagen types I and III, thereby leading to vascular stenosis and sclerosis [31].

In summary, S-nitrosylation exerts a dual regulatory effect on hypertension. On the one hand, it can relax blood vessels and reduce blood pressure; on the other hand, S-nitrosylation of some proteins may be related to the occurrence and maintenance of hypertension, such as promoting vascular sclerosis. Therefore, it is necessary to study more S-nitrosylated proteins and their effects on blood vessels and blood pressure.

### 2.2. Effect of Protein S-Nitrosylation in Pulmonary Arterial Hypertension

PAH is characterized by an elevated pulmonary vascular resistance and artery pressure, and its pathogenesis involves abnormal pulmonary vasoconstriction and pathological remodeling [32]. Excessive VSMC contraction and proliferation, as well as aberrant vascular endothelial cells, cause the pulmonary vascular lumen diameter to reduce and vascular stiffness to increase, thereby increasing vascular resistance. NO donor S-nitrosoglutathione (GSNO) can S-nitrosylate the α1-adrenoceptor to destroy the binding between the receptor and the ligand and effectively reduce the phenylephrine-induced contraction of the pulmonary artery ring [33]. S-nitrosylation also inhibits the 5-HT2 receptor system. Vasoconstriction in PAH that is mediated by 5-HT results in increased vascular resistance and elevated blood pressure. In isolated rabbit pulmonary artery rings, GSNO inhibited the 5-HT response by 77% in intact lungs and 82% in the pulmonary artery rings [34]. In addition to inhibiting the response of VSMCs to vasoactive substances, NO can directly S-nitrosylate actin to reduce the polymerization efficiency of G-actin and decrease vasoconstriction [19]. S-nitrosylated actin can act as a NO donor and exhibit rapidly effective vasodilator activity at unusually low concentrations. This may be a potential target for treating PAH.

**Figure 3:**
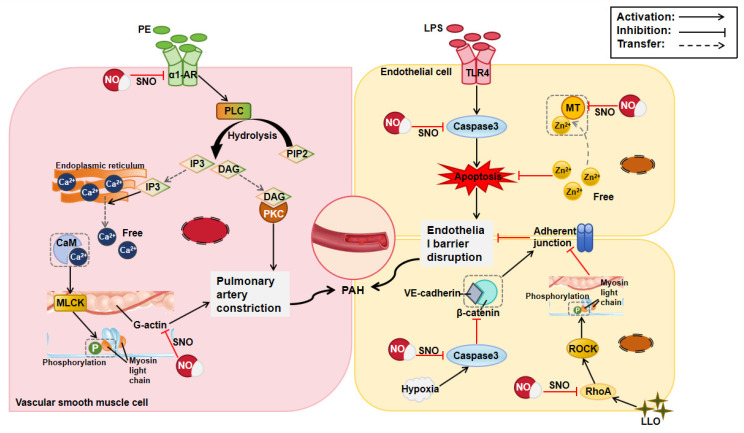
Mechanism of protein S-nitrosylation in pulmonary arterial hypertension (PAH). CaM: calmodulin, Caspase3: cysteine proteinase 3, DAG: diacylglycerol, IP3: inositol triphosphate, LLO: listeriolysin-O, LPS: lipopolysaccharide, MLCK: myosin light-chain kinase, MT: metallothionein, NO: nitric oxide, PE: phenylephrine, PIP2: phosphatidylinositol 4, 5-diphosphate, PKC: protein kinase C, PLC: phospholipase C, RhoA: Ras homolog gene family member A, ROCK: Rho-associated kinase, SNO: S-nitrosylation, TLR4: Toll-like receptor 4, VE-cadherin: vascular endothelial-cadherin, α1-AR: α1-adrenergic receptor.

Endothelial cell apoptosis and endothelial barrier disruption can exacerbate vascular remodeling and endothelial function loss, leading to an obvious increase in pulmonary vascular resistance. Therefore, protecting pulmonary artery endothelial cells (PAECs) from apoptosis and improving endothelial dysfunction may be important strategies for treating PAH. Studies have shown that NO promotes free zinc release in PAECs through S-nitrosylated metallothionein, thereby reducing the sensitivity of PAECs to LPS-induced apoptosis [20]. Similarly, prolonged NO synthesis by adenovirus-mediated iNOS transfer or NO donors’ SNAP administration inhibited LPS-induced apoptosis in sheep PAECs [35]. This inhibitory effect is closely related to NO suppressing the activation of IL-1β-converting enzyme-like cysteine protease (Caspase3-like) through S-nitrosylation, revealing the key protective role of S-nitrosylation in endothelial cells against LPS-induced apoptosis. The dysfunction of the vascular endothelial cell barrier is associated with its disrupted integrity by RhoA activation. NO donors can remarkably reduce RhoA activity that is elevated by Gram-positive bacterial toxins, thereby diminishing vascular permeability. This protective effect is achieved by RhoA S-nitrosylation at Cys16, Cys20, and Cys159, preventing RhoA from damaging the endothelial barrier [21]. Moreover, S-nitrosylation can regulate vascular permeability under hypoxia, which is also a factor affecting endothelial barrier function. SNAP inactivates caspase-3 via S-nitrosylation and loses its ability to cleave β-catenin, which maintains VE-cadherin complex formation and the adhesive junctions between the pulmonary microvascular endothelial cells, thereby preventing hypoxia-induced vascular leakage [22]. In conclusion, S-nitrosylation protects the barrier integrity of endothelial cells under hypoxia (Figure 3).

A feature of vascular remodeling in PAH is the enlarged vacuolated endothelial cells in pulmonary artery lesions [36]. The monocrotaline pyrrole (MCTP)-induced pulmonary artery endothelial megalocytosis model mimics this pathological feature. Low S-nitrosylation was observed in the endothelial cell membrane transport proteins including NSF, caveolin-1, and CHC, as well as the vascular-associated protein eNOS after MCTP treatment [37]. It is suggested that low protein S-nitrosylation may be related to the pathobiology of human PAH. Another piece of evidence is the lack of hemoglobin S-nitrosylation in PAH patients, which is associated with the effect of hypoxia on red blood cell (RBC) function, ultimately producing an excessive pulmonary vasoconstrictor response in vivo [38]. Grau et al. [39] found that NOS activation in RBCs was weakened following the deepening of hypoxia, which in turn affected the deformability of the RBCs, subsequently reducing blood flow, aggravating the burden on pulmonary vessels, and increasing the cardiac load of PAH patients. However, RBC deformability was surprisingly increased under severe hypoxia. This phenomenon was attributed to the reduction of nitrite to NO, which increased the S-nitration level of RBC α- and β-spectrins, maintaining RBC deformability. S-nitrosylation of hemoglobin is very important for tissue oxygenation. Cys within the β-chain of hemoglobin (βCys93) can retain the biological activity of NO in the blood after being modified to S-nitrosothiol by NO [40]. Once hypoxia leads to hemoglobin deoxygenation, erythrocytes release NO to glutathione (GSH) in the blood to form the vasodilator GSNO, thereby dilating blood vessels [41]. Decreased S-nitrosohemoglobin levels impair the vasodilator response to hypoxia and contribute to the development of PAH [42]. βCys93 alanine mutant mice also showed a systemic hypoxic response. On the other hand, inhibited βCys93 S-nitrosylation notably attenuated the short-term enhancement of the evoked respiratory response in mice during their recovery from hypoxia to normoxia [43]. Together, these studies suggest that S-nitrosylation may play an important role in maintaining a normal vascular response to hypoxia and during recovery.

Long-term PAH can trigger systemic hypoxia, and S-nitrosylation plays a key role in regulating the mechanism of pulmonary vascular response and cellular adaptation to hypoxic environments. Early studies have found that NOC-18, a NO donor, can enhance the DNA binding activity of HIF-1 by increasing HIF-1α and HIF-1β subunit expressions in bovine PAECs, thereby enhancing the cells’ adaptability to hypoxia [23]. Carver et al. [44] further found that in primary PAECs, GSNO increased the kinase activity of protein kinase B (Akt) in a dose- and time-dependent manner through inhibiting the counter-regulatory phosphatase PTEN via S-nitrosylation, thereby enhancing the stabilization and activation of downstream HIF-1α. Enhanced HIF-1 activity helps to promote angiogenesis, erythropoiesis, and vasodilation, thereby potentially attenuating some PAH pathological changes, such as pulmonary vascular remodeling and hypertension. Recent studies have found that acute hypoxia transiently increases the NO level in endothelial cells through activating eNOS, which further triggers protein S-nitrosylation. Chen et al. [45] demonstrated that the S-nitrosylation levels of at least 11 major proteins were obviously increased after acute hypoxia, including myosin (Cys 170) and β-actin (Cys 285), which verified the regulation of S-nitrosylation on pulmonary vascular response after hypoxia.

In summary, although PAH and systemic hypertension affect different vascular systems, their pathophysiological mechanisms are similar, with both characterized by abnormal vasoconstriction and vascular remodeling. Unlike the dual regulatory role of S-nitrosylation in hypertension, S-nitrosylation in PAH appears to have a more singular role, with the current research primarily emphasizing its protective effects (Table 1). On the one hand, protein S-nitrosylation in PAH inhibits vasoconstriction, protects endothelial cells, and modulates hypoxic responses. On the other hand, low levels of protein S-nitrosylation may be associated with vascular remodeling and impaired red blood cell function, thereby contributing to the development of PAH. Given the inadequacy of current PAH therapies in comprehensively covering its complex pathological mechanisms [46], further investigation into the specific mechanisms and regulatory pathways of S-nitrosylation is of significant importance for the development of therapeutic strategies for PAH.

### 2.3. Effect of Protein S-Nitrosylation in Atherosclerosis

Atherosclerosis is a chronic inflammatory arterial disease, and hyperhomocysteinemia (HHcy) is commonly used as a research model in experiments [47]. HHcy has been reported to promote the development of atherosclerosis by inhibiting vascular protein S-nitrosylation [48], suggesting an important regulatory role of protein S-nitrosylation in the atherosclerosis pathological process. Further studies have found that HHcy can reduce the Akt S-nitrosylation level via GSNO reductase (GSNOR)-dependent denitrosylation in T cells, thereby enhancing Akt-mediated T cell activation and inducing atherosclerosis [24]. Increased levels of S-nitrosylation appear to reduce vascular inflammation and oxidative stress, with subsequent improvement in atherosclerosis. In experiments on rabbits that were fed a high-cholesterol diet, the combination of probucol and cilostazol exerted a significant reduction in the atherosclerotic plaque area compared with the control group and showed antioxidant and anti-inflammatory effects, which are directly related to the obvious increase in plasma NO levels and protein S-nitrosylation levels in the endothelial cells by the combination treatment [49]. In addition, an experiment on human umbilical vein endothelial cells and ovariectomized rats showed that 17β-estradiol (E2β) increased eNOS phosphorylation and protein S-nitrosylation in vascular endothelial cells through the estrogen receptor α. E2β-induced S-nitrosylation prevented the AngII-stimulated upregulation of intercellular cell adhesion molecule-1 through a potential anti-inflammatory mechanism, contributing to vascular protection against atherosclerosis [50]. Moreover, E2β could downregulate the superoxide anion level and upgrade the protein S-nitrosylation level in VSMCs to combat the occurrence and development of atherosclerosis [51]. Thus, maintaining S-nitrosylation levels in vascular cells may have an atherogenic effect.

The massive accumulation of oxidized low-density lipoprotein (oxLDL) in the blood vessel intima can promote the process of atherosclerosis [52]. S-nitrosylation notably increases the hydrolytic activity of recombinant paraoxonase 1 (PON1), an enzyme responsible for many anti-atherogenic properties of high-density lipoprotein, and improves its ability to inhibit copper (II)-induced LDL oxidation [25,26]. However, once oxLDL has accumulated, iNOS may be activated and can subsequently increase S-nitrosylation of the molecular chaperone heat shock protein 90 (HSP90), leading to endothelial adhesion, inflammation, and oxidative stress in the endothelial cells [27]. S-nitrosylation of HSP90 inhibits its interaction with the activator of HSP90 ATPase activity 1 and eNOS activation, but a HSP90 mutation at Cys521 reverses these results and attenuates the atherosclerotic foci area. In hyperlipidemia, elevated plasma free fatty acids inhibit the ubiquitin-mediated degradation of integrin β1 in macrophages through iNOS-mediated S-nitrosylation of integrin β1, which facilitates the binding of integrin α4β1. The increased heterodimerization of integrin α4β1 leads to the enhanced adhesion of monocytes/macrophages to endothelial cells and vascular inflammation, thereby promoting the progression of atherosclerosis [28]. The rupture of unstable atherosclerotic plaques and stenosis or occlusion of the blood vessels lead to platelet aggregation and thrombosis, thereby triggering acute cardiovascular diseases [52]. NO inhibits ADP-induced platelet aggregation by S-nitrosylating up to 15 cysteine residues in tissue transglutaminase, especially at the Cys277 catalytic site, to maintain vascular homeostasis and prevent thrombosis and atherosclerosis [29]. NO can also inhibit the activity of protein disulfide isomerase by S-nitrosylating its catalytic active site, thereby reducing platelet aggregation and thrombosis [30].

In summary, during the formation stage or early phase of atherosclerosis, S-nitrosylation can protect the blood vessels and prevent the occurrence of atherosclerosis by regulating anti-inflammatory and antioxidant stress mechanisms. However, once atherosclerosis has already formed, the role of S-nitrosylation may change from a protective mechanism to one that promotes inflammation and oxidative stress, thereby driving the progression of the disease (Table 1). Exploring the selective modification of different proteins by S-nitrosylation at different stages of atherosclerosis and the underlying driving factors is conducive to grasping the precise timing of disease intervention to achieve the best therapeutic effect.

## 3. The Role of Protein S-Nitrosylation in Heart Diseases

### 3.1. Effect of Protein S-Nitrosylation in Heart Failure

**Figure 4:**
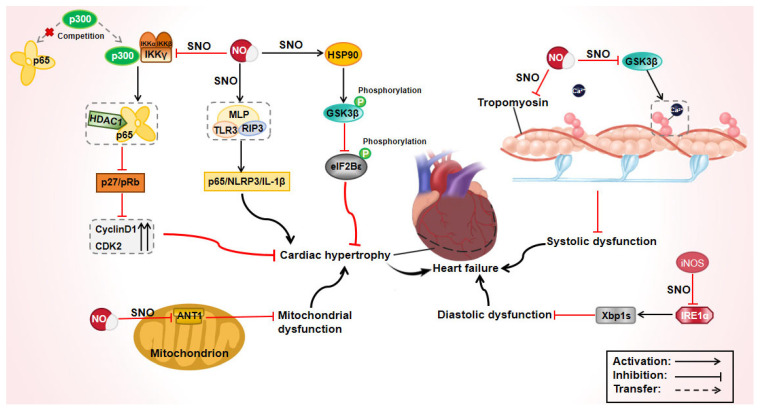
Mechanism of protein S-nitrosylation in heart failure. ANT1: adenine nucleotide translocase 1, CDK2: cyclin-dependent kinase 2, eIF2Bε: eukaryotic initiation factor 2B epsilon subunit, GSK-3β: glycogen synthase kinase-3β, HDAC1: histone deacetylase 1, HSP90: heat shock protein 90, IKKα, β, γ: IκB kinase α, β, γ, iNOS: inducible nitric oxide synthase, IL-1β: interleukin-1β, iNOS: inducible nitric oxide synthase, IRE1α: inositol-requiring enzyme 1α, MLP: muscle LIM protein, NF-κB: nuclear factor-kappa B, NLRP3: nod-like receptor protein 3, NO: nitric oxide, pRb: retinoblastoma protein; RIP3: receptor-interacting protein 3, SNO: S-nitrosylation, TLR4: Toll-like receptor 4, Xbp1s: X-box-binding protein 1.

Heart failure is a multifaceted syndrome resulting from myocardial damage, clinically categorized into heart failure with reduced ejection fraction (HFrEF) and heart failure with preserved ejection fraction (HFpEF) [53]. HFrEF is characterized by ventricular systolic dysfunction, with tropomyosin playing a pivotal role in regulating striated muscle contractions via Ca^2+^ [54]. In grade IV human heart failure, the S-nitrosylation level of tropomyosin in the left ventricle is significantly elevated compared to normal hearts [55]. Although this elevation is not directly linked to the left ventricular ejection fraction, the possibility that S-nitrosylation contributes to systolic dysfunction in end-stage heart failure cannot be excluded. Ventricular asynchrony in heart failure is associated with reduced Ca^2+^ sensitivity of myofilament proteins, and enhancing the activation energy of glycogen synthase kinase-3β (GSK-3β) improves this diminished reactivity, thereby enhancing contractile function [56]. However, S-nitrosylation inhibits GSK-3β activity, with nitrosylation/denitrosylation of the specific cysteine residues dynamically modulating GSK-3β function [57]. In a guinea pig model of heart failure, the S-nitrosylation levels of GSK-3β increased as the disease progressed, with S-nitrosylation-mediated inhibition eventually surpassing phosphorylation-induced activation of GSK-3β in the final stages [57]. This observation further supports the hypothesis that S-nitrosylation may play a role in systolic dysfunction in end-stage heart failure (Figure 4).

The clinical phenotype of HFpEF is notably complex, often associated with multiple comorbidities, and previous preclinical models have struggled to fully replicate the characteristics of HFpEF. Recently, Schiattarella et al. [58] developed a model that more accurately reproduces most of the clinical features of HFpEF by administering a high-fat diet and a NO synthase inhibitor L-NAME to mice. This model exhibited increased levels of iNOS and total protein S-nitrosylation, along with the inactivation of the inositol-requiring enzyme 1 alpha (IRE1α)/X-box binding protein 1 (Xbp1s) signaling pathway. Typically, IRE1α is activated and splices Xbp1s following phosphorylation, but S-nitrosylation inhibits IRE1α activity, leading to splicing defects and reduced Xbp1s in cardiomyocytes. In HFpEF mice, both iNOS knockout and Xbp1s overexpression partially improved diastolic dysfunction and heart failure symptoms. However, the therapeutic benefits of pharmacological iNOS inhibition in HFpEF appear to be independent of Xbp1s, suggesting alternative mechanisms at play. Further research using this model revealed that iNOS could also inhibit Akt activity by S-nitrosylation, thereby reducing glucose uptake by cardiomyocytes [59]. iNOS diminishes the insulin-mediated phosphorylation of Akt, while the pharmacological inhibition or genetic knockout of iNOS restores Akt phosphorylation and insulin signaling. Notably, iNOS does not affect basal Akt phosphorylation or glucose uptake in normal heart tissues. iNOS also plays a crucial role in myocardial fibrosis associated with HFpEF. An overexpression of iNOS in mouse cardiomyocytes increased the S-nitrosylation of PTEN, which in turn enhanced the phosphorylation of PI3K and AKT, leading to increased cell proliferation and migration, as well as the upregulated expression of collagen I and III [60]. These changes can be partially reversed by inhibiting iNOS or PI3K. In summary, iNOS plays a pivotal role in the pathophysiology of HFpEF, and targeting iNOS-mediated protein S-nitrosylation represents a promising therapeutic strategy for HFpEF (Figure 4).

In a failing heart, normal S-nitrosylation is disrupted by oxidative stress. Oxidative stress is recognized as a key pathophysiological factor in the onset and progression of heart failure [61]. In spontaneously hypertensive rats with heart failure, elevated xanthine oxidase activity generated excessive reactive oxygen species (ROS), which competed with S-nitrosylation to modify cysteine residues on the ryanodine receptor 2 (RyR2). This competition disrupted the normal S-nitrosylation of RyR2, resulting in heightened RyR2 activity and leading to Ca^2+^ leakage from the sarcoplasmic reticulum as well as impaired diastolic contractility [62]. Conversely, the increased expression of NADPH oxidase in the hearts of Duchenne muscular dystrophy mice enhanced the S-nitrosylation of connexin 43 (Cx43), although the specific mechanism remains unclear [63]. Excessive S-nitrosylation of Cx43 hemichannels heightens their activity, promoting cardiomyocyte apoptosis and accelerating cardiac remodeling and heart failure progression. Notably, the inhibition of NADPH oxidase restores the contractile response to β-adrenergic stimulation in isolated mouse hearts. Interestingly, in mice with heart failure induced by TAC, the S-nitrosylation of β-arrestin 2 was essential for maintaining the β-adrenergic response [64]. This suggests that the S-nitrosylation required for the β-adrenergic response in a failing heart may be inhibited by oxidative stress.

Cardiac hypertrophy represents an adaptive response to hemodynamic overload, aimed at maintaining cardiac function, but prolonged stress can lead to maladaptation and heart failure [65]. Muscle LIM protein (MLP) plays a critical role in stabilizing hypertrophic cardiomyopathy, and a *MLP* gene knockout in mice results in heart failure; however, MLP overexpression does not regulate cardiac hypertrophy [66,67]. This discrepancy arises because it is the S-nitrosylation on the MLP, rather than the MLP itself, that exerts the regulatory effects. S-nitrosylation of the MLP facilitates the formation of complexes with TLR3 and RIP3, activating the downstream p65/NLRP3/IL-1β pathway, which ultimately drives myocardial hypertrophy [68]. Similarly, S-nitrosylation of HSP90 enhances its interaction with GSK-3β, leading to increased GSK-3β phosphorylation and the reduced phosphorylation of its downstream target, eIF2Bε, thereby accelerating cardiac hypertrophy [69]. In TAC-induced cardiac hypertrophy models, the decreased expression of GSNOR correlates with the elevated S-nitrosylation levels of both the MLP and HSP90 [68,69]. Conversely, the overexpression of GSNOR reduces the S-nitrosylation levels of these proteins and remarkably alleviates hypertrophy symptoms. Therefore, GSNOR emerges as a critical target for mitigating nitrosative stress during myocardial hypertrophy, which is consistent with the research data that revealed that nitrosative stress in HFpEF was partly due to the impaired activity of GSNOR [70], as well as the reduced levels of GSNOR protein in myocardial samples from patients with hypertrophic cardiomyopathy [71]. By decreasing protein S-nitrosylation, GSNOR can also enhance cardiac myofilament sensitivity to Ca^2+^, thereby improving cardiac function [72]. Recently, Tang et al. [73] provided the first evidence that cytoplasmic GSNOR was transported to the mitochondrial stroma or intermembrane space via binding to HSP90 and translocase of outer mitochondrial membrane 70. The S-nitrosylation of adenine nucleotide translocase 1 (ANT1) impairs ANT1’s role in maintaining mitochondrial membrane potential and respiration, while mitochondrial GSNOR-mediated denitrosylation of ANT1 alleviates mitochondrial dysfunction in TAC-induced pathological cardiac hypertrophy and heart failure. During pathological cardiac hypertrophy, apoptosis and cell cycle activation are in opposition, regulated by p53 and p65, respectively. S-nitrosylated IκB kinase γ (IKKγ) enhances the interaction between p65 and p300 but has limited effects on the upregulation of cyclin D1 and CDK2 [74]. In contrast, non-nitrosylated IKKγ shows more significant upregulation. The arginine mutant of IKKγ at Cys410 competes with p53 and p65 for binding to p300, reducing apoptosis and promoting the binding of p65 to histone deacetylase 1. This leads to the deactivation of the CDK inhibitor p27 and the upregulation of phosphorylated pRb. Notably, inhibiting IKKγ S-nitrosylation downregulates NO release and reduces nitrosative stress.

In summary, in the different stages and types of heart failure, S-nitrosylation exacerbates disease progression by affecting the function of key proteins, thereby worsening pathological processes such as myocardial contractile dysfunction, myocardial fibrosis, apoptosis, and hypertrophy (Table 2). This is likely closely related to the oxidative stress that exists in the state of heart failure. Under oxidative stress, the S-nitrosylation of RYR2 is impaired, leading to calcium ion leakage and reduced contractility. Meanwhile, under oxidative stress, the excessive S-nitrosylation of Cx43 induces the apoptosis of cardiomyocytes and accelerates cardiac remodeling. Oxidative stress alters the degree of S-nitrosylation of different proteins, which may be a key factor affecting the selectivity of the S-nitrosylation of proteins, causing S-nitrosylation to shift from a protective role under physiological conditions to a detrimental role under pathological conditions. A deeper investigation into this complex interplay will facilitate the development of new strategies for intervening in the progression of heart failure.

### 3.2. Effect of Protein S-Nitrosylation on Myocardial Infarction

**Figure 5:**
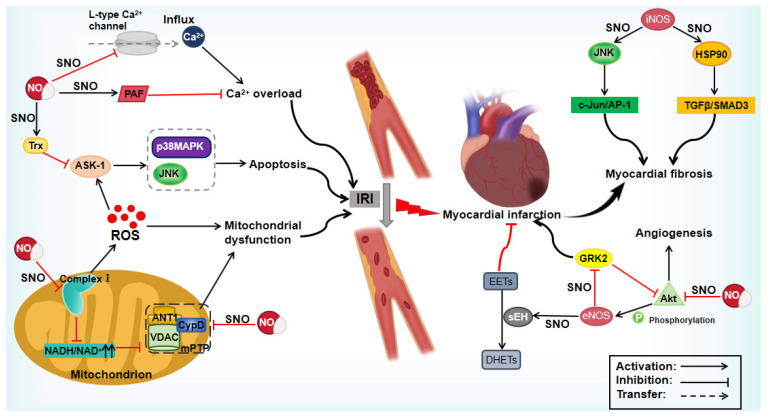
Mechanism of protein S-nitrosylation on myocardial infarction. Akt: protein kinase B, ANT1: adenine nucleotide translocase 1, AP-1: activator protein-1, ASK-1: apoptosis signal-regulating kinase 1, CypD: cyclophilin D, DHETs: dihydroxyeicosatrienoic acids, EETs: epoxyeicosatrienoic acids, eNOS: endothelial nitric oxide synthase, GRK2: G protein-coupled receptor kinase 2, HSP90: heat shock protein 90, iNOS: inducible nitric oxide synthase, IRI: ischemia–reperfusion injury, JNK: c-Jun NH2-terminal kinase, mPTP: mitochondrial permeability transition pore, NO: nitric oxide, PAF: platelet-activating factor, p38MAPK: p38 mitogen-activated protein kinase, ROS: reactive oxygen species, sEH: soluble epoxide hydrolase, SMAD3: SMAD family member 3, SNO: S-nitrosylation, TGF-β: transforming growth factor-β, Trx: thioredoxin, VDAC: voltage-dependent anion-selective channel.

Timely reperfusion is the preferred treatment following myocardial ischemia caused by acute myocardial infarction; however, this intervention can also lead to myocardial damage [97]. Therefore, preventing myocardial ischemia–reperfusion injury (MIRI) is of important clinical importance. The primary pathophysiological mechanisms underlying MIRI include apoptosis, intracellular Ca^2+^ overload, mitochondrial dysfunction, and oxidative stress [98]. S-nitrosylation can mitigate MIRI and reduce the size of myocardial infarction by interfering with these mechanisms (Table 2) (Figure 5). Apoptosis is a key contributor to MIRI, and blocking the signaling pathways that trigger apoptosis can clearly reduce the myocardial infarction size and enhance cardiac function [99]. Thioredoxin (Trx) is a ubiquitously expressed protein involved in various biological processes related to cell proliferation and apoptosis [100]. Trx directly interacts with apoptosis signal-regulating kinase 1 (ASK-1) to inhibit its activity and the subsequent activation of two pro-apoptotic kinases, p38 MAPK and c-Jun NH2-terminal kinase (JNK) [101]. Tao et al. [75] demonstrated that NO enhanced the inhibitory effect of Trx on p38 MAPK activity through S-nitrosylation, thereby protecting cardiomyocytes from I/R-induced apoptosis. During I/R, reducing Ca^2+^ overload can mitigate cardiac injury. Studies have shown that the S-nitrosylation of PAF, an inflammatory phospholipid, protects cells from I/R injury by decreasing intracellular Ca^2+^ overload [102]. Similarly, Sun et al. [103] indicated that NO could alter intracellular Ca^2+^ handling in cardiomyocytes, which was attributed to the reduction in L-type Ca^2+^ channel activity via S-nitrosylation.

Mitochondrial dysfunction is a key contributor to cell death and tissue injury associated with I/R injury during myocardial infarction [104]. A study demonstrated that the rapid reactivation of mitochondrial complex I is a key pathological feature of I/R injury, and that preventing this reactivation via S-nitrosylation provides cardioprotection [76]. MitoSNO, a mitochondrial-targeted S-nitrosylation donor, reversibly S-nitrosylates complex I, thereby slowing down the reactivation of mitochondria during the critical initial minutes of reperfusion. This process reduces ROS production, oxidative damage, and tissue necrosis. Interestingly, the inhibition of complex I is achieved through the selective S-nitrosylation of Cys39 on the ND3 subunit, which is specifically modified following ischemia. Consistent with a prior study, the pretreatment with SNO-MPG during ischemia can induce the S-nitrosylation of mitochondrial proteins, safeguarding crucial thiol groups in these proteins from irreversible oxidative damage [105]. The S-nitrosylation of complex I increases NADH levels, leading to an elevated mitochondrial GSH/GSSG ratio, which enhances GSH’s capacity to detoxify peroxides. Therefore, the inhibition of Complex I oxidation may be a potential mechanism underlying S-nitrosylation-mediated cardioprotection. Additionally, ischemic preconditioning increases protein S-nitrosylation in cardiomyocytes, with most proteins showing reduced or no oxidation at the same sites following ischemia and early reperfusion [106]. This indirectly supports the notion that rapid and reversible protein S-nitrosylation exerts cardioprotective effects by protecting cysteine residues from oxidative stress induced by reactive oxygen species. A recent study has identified Stanniocalcin 1 (Stc1) as a functional factor in hypoxia-preconditioned cardiomyocytes. After being secreted into the microenvironment, Stc1 activates the calcium-sensing receptor, thereby increasing STAT3 phosphorylation via iNOS-mediated S-nitrosylation, and ultimately reducing cardiomyocyte apoptosis/pyroptosis. The pericardial delivery of recombinant Stc1 encapsulated in a hydrogel extended the therapeutic time window of recombinant Stc1 and improved long-term cardiac function [107]. This approach may represent a feasible alternative to ischemic preconditioning in clinical settings.

S-nitrosylation exerts beneficial effects on hypoxic myocardial tissue. Nitroglycerin (NTG), a potent vasodilator, can induce the S-nitrosylation of the mitochondrial protein cyclophilin D (CypD), inhibiting the opening of the CypD-mediated mPTP, and thus limiting myocardial infarction size [77]. The S-nitrosylation effect of NTG on CypD is dependent on eNOS. However, Ding et al. [78] found that NO effectively induced S-nitrosylation of soluble epoxide hydrolase (sEH) during the early stages of reperfusion, which enhanced the metabolism of EETs to less potent DHETs, exacerbating heart I/R damage. In this context, the deletion of eNOS inhibited sEH activation. In both studies, mouse models were reperfused for 2 h after 30 min of ischemia, while a continuous infusion of NTG at 24 μg/kg/min for 65 min, starting from 20 min into ischemia, obviously reduced infarct size. Thus, NO application is generally beneficial for myocardial infarction. However, long-term NTG treatment may induce tolerance. Sayed et al. [108] demonstrated that in vivo nitrate tolerance was partially mediated by NTG-dependent sGC desensitization. In the primary aortic smooth muscle cells, S-nitrosylation reagent CSNO treatment caused S-nitrosylation at Cys243 of the α subunit and Cys122 of the β subunit of sGC, decreasing the responsiveness of sGC to NO in a concentration- and time-dependent manner [79]. Similarly, in mice with an endothelial-specific overexpression of eNOS, the total amount of sGC protein was unchanged, but its activity was inhibited by S-nitrosylation [109]. Actually, in normal physiological conditions, NO inhibits sGC activity through S-nitrosylation to prevent its overactivation. This negative feedback maintains the stability of the NO–sGC–cGMP signaling pathway. However, long-term NTG treatment increases sGC S-nitrosylation, reducing its sensitivity to NO and causing tolerance. Additionally, long-term NTG treatment can reduce Akt activity through S-nitrosylation, leading to the delayed recovery of cardiac function and impaired angiogenesis in patients with ischemic heart disease [80]. The expression of mutated Akt at Cys296 and Cys344 accelerated cardiac function recovery in NTG-treated mice post-myocardial infarction. GPCR kinase 2 (GRK2), a major effector of myocardial cell death after ischemia, can directly bind to Akt, reducing Akt-mediated phosphorylation of eNOS [81]. GRK2 itself can be modified by eNOS via S-nitrosylation, thereby reducing ischemic heart damage. Therefore, long-term NTG treatment may not only lead to drug tolerance but also exacerbate heart function. In conclusion, short-term, acute, small-dose NTG administration before reperfusion may be clinically recommended.

During myocardial infarction, the ischemic death of a large number of cardiomyocytes activates repair mechanisms, where fibroblasts and myofibroblasts form scars at the injury site. However, excessive fibrosis and reactive fibrosis at non-injured sites can gradually impair cardiac function [110]. Research has shown that the S-nitrosylation level of HSP90 in cardiac fibroblasts from patients with cardiac fibrosis is elevated and positively correlated with the fibrosis marker, alpha-smooth muscle actin [82]. S-nitrosylation of HSP90 activates the TGFβ/SMAD3 signaling pathway by binding to the TGFβII receptor, thereby contributing to myocardial fibrosis development. In this process, iNOS serves as the source of endogenous NO. Both iNOS inhibition and mutations in HSP90 at Cys589 can prevent cardiac fibrosis in TAC model mice. The mechanism by which iNOS drives fibrosis is multifaceted. iNOS can also induce the activation of JNK through S-nitrosylation, leading to the expression of pro-fibrosis genes [83]. The S-nitrosylation of JNK at Cys116 and Cys163 can induce myocardial fibroblast differentiation by activating the c-Jun/AP-1 pathway. The knockout of iNOS prevented cardiac fibrosis in TAC model mice by reducing the S-nitrosylation JNK. Without timely intervention, myocardial fibrosis can eventually lead to heart failure. The treatment of cardiac fibrosis remains a significant challenge, but iNOS represents a promising therapeutic target.

In summary, S-nitrosylation shows benefits in myocardial infarction treatment by inhibiting apoptosis signaling, reducing calcium overload, and preventing mitochondrial dysfunction, thus protecting against ischemia–reperfusion injury. However, the role of S-nitrosylation in myocardial infarction treatment depends on various factors, such as the target proteins, the timing of modification, and its duration. Long-term NTG treatment can disrupt S-nitrosylation balance, causing tolerance and potential damage. For example, the S-nitrosylation of sGC is a negative feedback mechanism that helps maintain sGC activity at an appropriate level. However, excessive S-nitrosylation can desensitize sGC to NO. Given the crucial role of the NO–sGC–cGMP signaling pathway in vascular homeostasis, sGC desensitization may also lead to adverse effects beyond NTG tolerance. Therefore, in clinical practice, it is essential to carefully evaluate the potential risks associated with S-nitrosylation modification to achieve optimal therapeutic outcomes.

### 3.3. Effect of Protein S-Nitrosylation on Arrhythmia

The regulatory effect of S-nitrosylation on arrhythmia primarily relies on the modification of myocardial ion channels by nNOS-derived NO. The nNOS adaptor protein (nNOSAP) has been discovered to colocalize with L-type Ca^2+^ channels and the potassium (K^+^) channel Kir3.1 in cardiomyocytes. The inhibition of nNOSAP expression was associated with reduced Ca^2+^ influx and decreased S-nitrosylation, suggesting that nNOSAP plays a critical role in modulating cardiac action potential and repolarization via S-nitrosylation [111]. nNOS is a Ca^2+^-dependent enzyme that interacts with the cardiac Ca^2+^ release channels RyR and SERCA2 [112]. SERCA2 is essential in maintaining Ca^2+^ homeostasis within cardiomyocytes. Burger et al. [84] observed a higher incidence of ventricular arrhythmias in nNOS^−/−^ mice compared to wild-type mice post-myocardial infarction. The absence of nNOS resulted in an increased Ca^2+^ transient amplitude, heightened L-type Ca^2+^ channel activity, and elevated diastolic Ca^2+^ levels in cardiomyocytes. Post-myocardial infarction, the S-nitrosylation levels of the L-type Ca^2+^ channels, RyR2 and SERCA2, were diminished. This suggests that nNOS may confer cardioprotective effects by regulating the S-nitrosylation of Ca^2+^ handling proteins and inhibiting L-type Ca^2+^ channel activity, thus reducing the risk of ventricular arrhythmias and mortality following myocardial infarction. The EF-hand type Ca^2+^ sensor protein S100-A1, which interacts with both RyR2 and SERCA2, exerts positive inotropic and antiarrhythmic effects in cardiomyocytes [113]. Under physiological conditions, S100-A1 undergoes endogenous S-nitrosylation at Cys85, which optimizes its conformation for Ca^2+^ binding [85]. Without this modification, S100-A1 exhibits a low Ca^2+^ affinity, hindering its interaction with RyR2 and SERCA2. A recent study has indicated that Cys85 of S100-A1 may function as a redox switch, which is essential for the antiarrhythmic effects of S100A1 in cardiomyocytes but not for its positive inotropic actions. The S-nitrosylation of this site determines the beneficial regulatory capacity of S100-A1 over diastolic, but not systolic, RyR2 activity [86]. These observations indicate that the S-nitrosylation of RyR2 and SERCA2 is advantageous in maintaining a normal cardiac rhythm. However, in isoproterenol-induced arrhythmias, S-nitrosylation exerts a dual effect. S-nitrosylation of different sites on CaMKIIδ either inhibits or enhances its activation from the calmodulin complex, thereby regulating the phosphorylation activation of RyR2 and Ca^2+^ leakage [87]. The factors influencing site selectivity are still unknown. However, GSNO pretreatment can reduce isoproterenol-induced arrhythmias, whereas GSNO administration after increased β-AR stress increases the frequency of arrhythmias. This may be due to steric hindrance or the allosteric effects between the sites on CaMKIIδ that are S-nitrosylated and those that are acted upon by the calmodulin complex.

During the cardiac action potential plateau, the inward rectifier potassium channels exhibit relatively weak ionic conductance, permitting only a minimal inward current to sustain the plateau phase. However, during the repolarization phase, these channels facilitate a considerable K^+^ outflow, which helps to restore the cell to its resting membrane potential [114]. Among the inward rectification channels, Kir2.1 channels represent the predominant subtype in the heart [115]. Gómez et al. [88] demonstrated that the physiological levels of NO enhanced the probability of Kir2.1 channel opening via S-nitrosylation, thereby increasing I_Kir2.1_ and reducing arrhythmia incidence. These findings offer deeper insights into the role of I_K1_ in cardiac excitability regulation. The cardiac Na^+^ channel, a macromolecular complex, can undergo S-nitrosylation, which enhances Na^+^ conduction in response to NO [116]. Dallas et al. [89] found that CO induced early afterdepolarizations by stimulating nNOS in cardiomyocytes, leading to elevated local NO levels. This, in turn, resulted in S-nitrosylation of the Na^+^ channel protein Nav1.5, thereby increasing late Na^+^ current (I_Na_) conduction. These findings suggest that nNOS-mediated NO synthesis and excessive NO release in cardiomyocytes can enhance I_Na_ and that the S-nitrosylation of cardiac Na^+^ channels may contribute to arrhythmogenesis. Caveolin-3 exerts a key inhibitory effect on late I_Na_. Cheng et al. [90] observed that the co-expression of caveolin-3 mutations with Nav1.5 leads to an increase in I_Na_. These effects arise because caveolin-3 mutations diminish nNOS inhibition, locally increase the direct S-nitrosylation of Nav1.5, and substantially prolong action potential duration and delay repolarization. These results suggest that S-nitrosylation of the Na^+^ channel protein Nav1.5 promotes late I_Na_ production and that inhibiting S-nitrosylation could normalize I_Na_ and prevent heart failure or ischemic arrhythmias. In contrast to caveolin-3, myocardial caveolin-1 is correlated with left ventricular conduction velocity, and mice with caveolin-1 deficiency exhibit a heightened susceptibility to ventricular arrhythmias [91]. S-nitrosylation of caveolin-1 disrupts its interaction with c-Src, leading to c-Src dissociation and activation. This, in turn, results in the diminished expression of Cx43, which impairs gap junction functionality, thereby reducing the ventricular conduction velocity and increasing the predisposition to ventricular arrhythmias.

**Figure 6:**
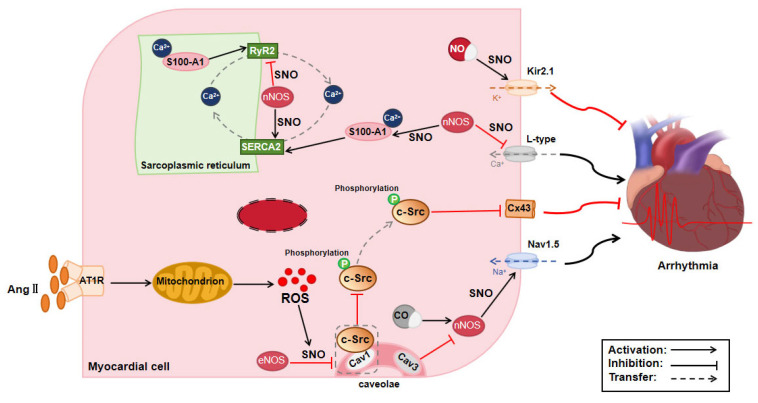
Mechanism of protein S-nitrosylation on arrhythmia. AngII: angiotensin II, AT1R: angiotensin type 1 receptor, Cav1, 3: Caveolin 1, 3, CO: carbon monoxide, Cx43: connexin 43, eNOS: endothelial nitric oxide synthase, NO: nitric oxide, ROS: reactive oxygen species, RyR2: ryanodine receptor 2, SERCA2: sarcoplasmic/endoplasmic reticulum calcium ATPase 2, SNO: S-nitrosylation, nNOS: neuronal nitric oxide synthase.

In summary, nNOS-dependent S-nitrosylation modulates cardiac action potential and repolarization by modifying ion channels on the cardiomyocyte membrane, thereby influencing the occurrence of arrhythmias. nNOS can S-nitrosylate L-type Ca^2+^ channels, Kir3.1 potassium channels, and Nav1.5 sodium channels, regulating their activities and thus affecting cardiac electrophysiological properties (Table 2) (Figure 6). For instance, S-nitrosylation of Kir2.1 increases channel open probability, enhancing inward rectifier potassium currents and reducing arrhythmias. Conversely, S-nitrosylation of Nav1.5 increases late I_Na_, leading to arrhythmias. The integrated regulation of multiple ion channels, including Ca^2+^, K^+^, and Na^+^ channels, by nNOS may play a complex role in arrhythmogenesis. Future research should further elucidate the specific mechanisms to identify new therapeutic targets for arrhythmias.

### 3.4. Effect of Protein S-Nitrosylation in Diabetic Cardiomyopathy

Obesity and diabetes are linked to an increased vulnerability to heart I/R injury, and the cardioprotective benefits of regular exercise may be attributed to then enhanced activation of β3AR and eNOS. Kleindienst et al. [117] developed a mouse model of obesity and diabetes to observe myocardial I/R responses. The study revealed that obese and diabetic mice were more susceptible to I/R damage, both in vivo and in vitro, and exhibited more severe heart failure symptoms due to the dysfunction of the β3AR/eNOS pathway in their hearts. Hyperglycemia-induced metabolic stress increases iNOS levels and nitrosative stress, which in turn inhibits AMPK activation and its promotion of collateral circulation in the coronary arteries through S-nitrosylation [92]. During early reperfusion, exercise restored normal iNOS levels in these diseased mice and reduced protein S-nitrosylation, nitrotyrosination, and ROS production. Further investigations showed that even with defects in the β3AR/eNOS pathway, regular exercise protected the hearts of the diseased mice from I/R injury by decreasing iNOS expression and nitrosative stress. Okazaki et al. [93] demonstrated that the administration of the NOS co-factor tetrahydrobiopterin (BH4) prior to heart I/R in diabetic rats led to the NO-mediated S-nitrosylation of BH4, which reduced iNOS-derived superoxide production and consequently improved left ventricular function.

The diabetic myocardium is more prone to I/R injury due to increased oxidative stress, while S-nitrosylation protects cardiac cells from oxidative stress. The nuclear factor erythroid 2-related factor 2 (Nrf2) is a critical regulator of cellular oxidative stress, controlling the expression of antioxidant genes by translocating to the nucleus and binding to antioxidant response elements [118]. Kelch-like ECH-associated protein 1 (Keap1) acts as a negative regulator of Nrf2. Xiao et al. [94] found that the polyphenolic compound luteolin induced the S-nitrosylation of Keap1 via eNOS activation, which in turn activated Nrf2 and promoted its nuclear translocation, thereby inhibiting oxidative stress and reducing diabetic myocardial I/R injury. However, in diabetes, the upregulation of MAP4K4 promotes the S-nitrosylation of Drp1, a key regulator of mitochondrial dynamics, by inhibiting the expression of the antioxidant enzyme GPX4. This chain of events stimulates ferroptosis in cardiac microvascular endothelial cells, ultimately leading to microcirculatory dysfunction in diabetic cardiomyopathy [95]. These findings suggest interregulation between oxidative stress and S-nitrosylation. Furthermore, Fillmore et al. [96] demonstrated that S-nitrosylation of tripartite motif-containing 72 (TRIM72) by NO conferred cardioprotective effects in mice with myocardial I/R injury. TRIM72, a membrane repair protein predominantly expressed in the heart and skeletal muscle, is degraded during I/R injury, thereby losing its ability to act as a scaffold for membrane repair. Under oxidative stress conditions in HEK293 cells, S-nitrosylation at Cys144 protects TRIM72 from oxidative degradation. Collectively, these findings indicate that S-nitrosylation alleviates oxidative stress and thereby improves diabetic cardiomyopathy (Table 2).

In summary, cardiomyocytes in the diabetic state exhibit a decreased tolerance to I/R due to increased oxidative stress, while S-nitrosylation mitigates diabetic myocardial I/R injury by attenuating oxidative stress. However, a reciprocal regulatory relationship exists between oxidative stress and S-nitrosylation. Oxidative stress can also modulate the levels of S-nitrosylation, for instance, by upregulating iNOS expression to augment NO production, thereby influencing the S-nitrosylation of specific proteins. Further elucidation of the interplay between oxidative stress and S-nitrosylation may offer novel therapeutic strategies for I/R injury in diabetic cardiomyopathy.

## 4. Conclusions and Prospect

This paper examines the role of protein S-nitrosylation in various heart diseases, including systemic hypertension, PAH, atherosclerosis, heart failure, myocardial infarction, arrhythmia, and diabetic cardiomyopathy. S-nitrosylation of the transcription cofactor myocardin attenuates the phenotypic marker expression of VSMC contractility and vasoconstriction in systemic hypertension. S-nitrosylation can also reduce the response of VSMCs to vasoactive substances, including AngII and epinephrine released by sympathetic overactivation, by acting on GPCRs. However, increased levels of protein S-nitrosylation in adventitial fibroblasts can promote the excessive deposition of type I and type III collagen, leading to vascular stenosis and stiffness. In PAH, S-nitrosylation of α1-adrenoceptors and 5-HT2 receptors contributes to vasodilation. S-nitrosylation of metallothionein and RhoA in PAECs protects endothelial cells from apoptosis and endothelial barrier disruption, respectively. Additionally, S-nitrosylation of hemoglobin and HIF-1 helps regulate pulmonary artery responses and improve oxygenation, aiding the body in coping with the hypoxic environment caused by PAH. In atherosclerosis, the increased levels of protein S-nitrosylation in blood vessels exert anti-inflammatory, anti-oxidative, and anti-oxLDL effects, thereby improving atherosclerosis. S-nitrosylation has been implicated in both systolic and diastolic dysfunction in heart failure. The elevated S-nitrosylation levels of tropomyosin and GSK-3β in HFrEF are associated with systolic dysfunction in advanced heart failure. In HFpEF, iNOS-driven increases in the S-nitrosylation levels of IRE1α and Akt contribute to diastolic dysfunction and reduced glucose uptake in cardiomyocytes. Markedly elevated S-nitrosylation levels of the MLP and HSP70 are associated with the progression of cardiac hypertrophy in TAC-induced models. S-nitrosylation of ANT1 is implicated in cardiomyopathic hypertrophy and mitochondrial dysfunction during heart failure. In myocardial infarction-related I/R models, S-nitrosylation of Trx, PAF, GRK2, CypD, and the ND3 subunit of mitochondrial complex I reduces infarct size and protects the heart from I/R injury, while S-nitrosylation of sEH exacerbates I/R damage. In TAC-induced myocardial fibrosis, increased S-nitrosylation levels of HSP90 and JNK, mediated by iNOS, promote the development of myocardial fibrosis. S-nitrosylation of caveolin-1 heightens the risk of ventricular arrhythmia, whereas S-nitrosylation of RyR2, SERCA2, S100-A1, and L-type Ca^2+^ channels supports cardiac rhythm stability. S-nitrosylation of Nav1.5 may induce arrhythmias, while S-nitrosylation of Kir2.1 appears to mitigate arrhythmogenic risk. S-nitrosylation of BH4 decreases iNOS-derived superoxide production, thereby enhancing left ventricular function. The weakened binding of Keap1 to Nrf2 following S-nitrosylation promotes Nrf2 nuclear translocation and the upregulation of antioxidant genes, reducing oxidative stress and mitigating diabetic myocardial I/R injury. S-nitrosylation of TRIM72 safeguards it from oxidative degradation, thus protecting the heart. In summary, the effects of S-nitrosylation are context-dependent, varying with the specific disease and protein involved, and can have either protective or detrimental outcomes. Modulating specific protein S-nitrosylations could represent a promising therapeutic strategy for managing cardiac disease.

By summarizing the above studies, it can be found that inflammation, oxidative stress, and metabolic factors all contribute to the upregulation of iNOS in the cardiovascular system. Moreover, iNOS-mediated protein S-nitrosylation generally exacerbates pathological changes associated with cardiovascular diseases. Although all NOS can produce NO and induce protein S-nitrosylation, the activation of eNOS and nNOS is usually cardioprotective. This may be because iNOS irreversibly binds to calmodulin, resulting in its continuous activation and the rapid, excessive production of NO. iNOS has a relatively simple structure, lacking an autoinhibitory domain. Additionally, mutations in its calmodulin-binding region enable it to bind tightly to calmodulin even at low Ca^2+^ concentrations, and once bound, it is difficult to dissociate [119]. This Ca^2+^-independent activity contrasts with that of eNOS and nNOS, which can dynamically regulate their activity and NO production in response to intracellular Ca^2+^ levels through binding and dissociation with calmodulin. Therefore, in pathological states such as inflammation, iNOS may cause cardiovascular damage through persistent and excessive protein S-nitrosylation. However, the regulatory mechanisms of iNOS activity are complex, and appropriate modulations can also exert cardioprotective effects. Recent reports have shown that transgenic mice overexpressing valosin-containing protein (VCP), an ATPase-associated protein that protects the heart from cardiac stress and ischemic injury, exhibit increased global protein S-nitrosylation levels. Knockout of iNOS in these mice eliminated the S-nitrosylation of mitochondrial complex proteins, suggesting that VCP attenuates oxidative stress-related cardiac injury by modulating iNOS [120]. Furthermore, there is a scarcity of recent literature on NO-induced protein S-nitrosylation in vascular tissues. Increasing evidence suggests that the role of NO in the cardiovascular system is independent of the sGC/cGMP signaling pathway and is instead mediated by S-nitrosylation. Therefore, it is necessary to re-evaluate previous studies to explore potential S-nitrosylation mechanisms. For instance, the reduction in ET-1 secretion in endothelial cells induced by NO, which is independent of cGMP, may imply the involvement of S-nitrosylation [15].

Despite the identification of over 3000 S-nitrosylated proteins and more than 4000 S-nitrosylation sites [121], in vivo detection and quantification of protein S-nitrosylation remain limited due to the lack of highly sensitive and specific methods [122,123]. The instability of S-nitrosylation and its dynamic nature, which varies with time and cellular state, further complicate accurate quantification. The high interindividual variability in the transient and sensitivity of metabolic fluxes makes it difficult to obtain consistent results when detecting and quantifying S-nitrosylation in vivo, which may affect the reproducibility of the test results. Similarly, technical limitations lead to the existence of many S-nitrosylation targets that have not yet been identified and characterized. These challenges, coupled with other unresolved issues that include the lack of comprehensive prediction and understanding of S-nitrosylation sites, the unresolved specificity and selectivity of S-nitrosylation, the unclear mechanisms by which S-nitrosylation protects cardiomyocytes through the regulation of mitochondrial function and calcium homeostasis, and the unelucidated interactions between S-nitrosylation and other post-translational modifications (such as phosphorylation and ubiquitination), as well as their integrated regulation of protein function, highlight ongoing research needs. Among the identified targets, HSP90, Ca^2+^ channels, and mitochondrial complex I show the most promise for therapeutic intervention. On the one hand, various cardiovascular diseases involve increases in iNOS, intracellular Ca^2+^ levels, and oxidative stress, while HSP90, Ca^2+^ channels, and mitochondrial complex I are each relevant regulatory proteins. On the other hand, these proteins all hold potential for clinical translation. Mitochondria-targeted S-nitrosylating agents were developed over a decade ago and have shown protective effects in cardiovascular diseases in preclinical experiments. At present, more than 10 inhibitors of HSP90 have entered clinical trials, and Ca^2+^ channel inhibitors such as calcium antagonists have been widely used in clinical practice [124]. Although these inhibitors are not related to post-translational modifications, the role of S-nitrosylation in regulating the activity of key proteins is analogous to that of inhibitors, providing a foundation and the confidence for developing drugs targeting S-nitrosylation.

Modulating the S-nitrosylation of specific proteins to interfere with disease progression may represent a promising therapeutic strategy. For example, Kar et al. [125] successfully downregulated nitrosative stress and apoptosis, reduced infarct size, and improved the pathophysiology of myocardial infarction by selectively delivering an S-nitrosylated mutant of NEMO (IKKγ) (R-NEMO) via a targeting chitosan nanoparticle to the affected cardiomyocytes. Moreover, a newly developed small-diameter artificial blood vessel graft based on silk fibroin (NOeGraft) promotes the generation of the bioactive gas NO through S-nitrosylation, presenting a novel pathway for the clinical translation of S-nitrosylation [126]. Overall, the effects of S-nitrosylation vary depending on the disease type and the specific proteins involved. However, in general, it appears to be beneficial.

## Figures and Tables

**Table 1:** Role and site of protein S-nitrosylation in vascular diseases.

Disease	Protein	Site	Effect	Reference
Systemic hypertension	Myocardin	C596	Reduced the expression of contractile markers	[10]
	PKC	-	Inhibited vasoconstriction	[11]
	AT1R	C289	Decreased AT1R’s binding affinity for AngII	[12]
	Cav1.2 channel	C1180, C1280	Limited Ca^2+^ influx and vasoconstriction	[13]
	STIM2	C15, C53, C60	Reduced intracellular Ca^2+^ concentration and vasoconstriction	[14]
	CaMKIIγ	-	Inhibited α1-adrenergic vasoconstriction	[16]
	β-arrestin 2	C410	Accelerated β-adrenergic receptor endocytosis	[17]
	RhoA	C16, C20	Inhibited vasoconstriction	[18]
Pulmonary arterial hypertension	Actin	C374	Promoted vasodilation	[19]
	MT	-	Reduced pulmonary artery endothelial cell apoptosis	[20]
	RhoA	C16, C20, C159	Reduced pulmonary vascular endothelial barrier destruction	[21]
	Caspase3	C163	Maintained adherent junctions of pulmonary vascular endothelial cells	[22]
	HIF-1	-	Promoted angiogenesis and improved oxygenation	[23]
Atherosclerosis	Akt	C224	Reduced T cell inflammation and atherosclerosis	[24]
	PON1	C284	Inhibited LDL oxidation and atherosclerosis process	[25,26]
	HSP90	C521	Aggravated atherosclerosis	[27]
	Integrin β1	C555	Promoted vascular inflammation and atherosclerosis progression	[28]
	tTG	C277	Inhibited platelet aggregation and thrombosis	[29]
	PDI	C397, C400	Reduced platelet aggregation and thrombosis	[30]

Abbreviations: Akt: protein kinase B, AngII: angiotensin II, AT1R: angiotensin type 1 receptor, CaMKIIγ: calcium/calmodulin-dependent protein kinase II γ, Caspase3: cysteine proteinase 3, HIF-1: hypoxia inducible factor-1, HSP90: heat shock protein 90, LDL: low density lipoprotein, MT: metallothionein, NO: nitric oxide, PDI: protein disulfide isomerase, PKC: protein kinase C, PON1: paraoxonase 1, RhoA: Ras homolog gene family member A, sGC: soluble guanylyl cyclase, STIM2: stromal interaction molecule-2, tTG: tissue transglutaminase.

**Table 2:** Role and site of protein S-nitrosylation in cardiac diseases.

Disease	Protein	Site	Effect	Reference
Heart failure	Tropomyosin	-	Induced systolic dysfunction	[55]
	GSK-3β	C76, C178, C199, C245, C317, C335	Induced systolic dysfunction	[57]
	IRE1α	-	Induced diastolic dysfunction	[58]
	Akt	C224	Decreased myocardial glucose uptake	[59]
	RyR2	-	Improved diastolic SR Ca^2+^ leakage and decreased contractility	[62]
	Cx43	-	Induced cardiomyocyte apoptosis and accelerated heart failure	[63]
	β-arrestin 2	C253	Maintained β-adrenergic response	[64]
	MLP	C79	Myocardial hypertrophy occurred and developed	[68]
	HSP90	C589	Accelerated cardiac hypertrophy	[69]
	ANT1	C160	Myocardial cell mitochondrial dysfunction occurred	[73]
	IKKγ	C410	Induced cardiomyocyte apoptosis and cardiac hypertrophy	[74]
Myocardial infarction	Trx	C69	Inhibited ASK-1 proapoptotic effect and reduced MIRI	[75]
	Complex I ND3 subunit	C39	Reduced ROS production and alleviated MIRI	[76]
	CypD	-	Inhibited mPTP opening and limited myocardial infarction area	[77]
	sEH	C141	Aggravated MIRI	[78]
	sGC	C122, C243	Decreased the response of sGC to NO	[79]
	Akt	C296, C344	Impaired angiogenesis	[80]
	GRK2	C340	Reduced ischemic heart damage	[81]
	HSP90	C589	Promoted cardiac fibrosis	[82]
	JNK	C116, C163	Induced myocardial fibroblast differentiation	[83]
Arrhythmia	L-type Ca^2+^ channel RyR2 SERCA2	-	Reduced ventricular arrhythmias after myocardial infarction	[84]
	S100-A1	C85	Induced positive inotropic action and anti-arrhythmia	[85,86]
	CaMKIIδ	C273, C290	Limited or worsened β-adrenergic receptor-induced arrhythmias	[87]
	Kir2.1	C76	Increased Kir2.1 channel opening and reduced arrhythmias	[88]
	Nav1.5	-	Promoted late I_Na_ and arrhythmia	[89,90]
	Caveolin-1	C156	Increased risk of ventricular arrhythmia	[91]
Diabetic cardiomyopathy	AMPKγ1	C130	Disrupted coronary collateral circulation	[92]
	BH4	-	Reduced iNOS-derived superoxide and improved left ventricular function	[93]
	Keap1	-	Inhibited oxidative stress and reduced diabetic MIRI	[94]
	Drp1	C650	Promoted endothelial dysfunction in diabetic cardiomyopathy	[95]
	TRIM72	C144	Reduced degradation of TRIM72 and protected the heart	[96]

Abbreviation: Akt: protein kinase B, AMPKγ1: AMP-activated protein kinase γ 1, ANT1: adenine nucleotide translocase 1, ASK-1: apoptosis signal-regulating kinase 1, BH4: tetrahydrobiopterin, CaMKIIδ: calcium/calmodulin-dependent protein kinase II δ, Cx43: connexin 43, CypD: cyclophilin D, Drp1: dynamin-related protein 1, GRK2: G protein-coupled receptor kinase 2, GSK-3β: glycogen synthase kinase-3β, HSP90: heat shock protein 90, IKKγ: IκB kinase-γ, iNOS: inducible nitric oxide synthase, IRE1α: inositol-requiring enzyme 1α, JNK: c-Jun NH2-terminal kinase, Keap1: Kelch-like ECH-associated protein 1, MIRI: myocardial ischemia–reperfusion injury, MLP: muscle LIM protein, mPTP: mitochondrial permeability transition pore, ROS: reactive oxygen species, RyR2: ryanodine receptor 2, SERCA2: sarcoplasmic/endoplasmic reticulum calcium ATPase 2, sEH: soluble epoxide hydrolase, SR: sarcoplasmic reticulum, TRIM72: tripartite motif-containing protein 72, TrX: thioredoxin, tTG: tissue transglutaminase.

## Data Availability

Not applicable.

## Abbreviations

The following abbreviations are used in this manuscript:

ADP	adenosine diphosphate
AMPK	AMP-activated protein kinase
AP-1	activator protein-1
Caspase3	cysteine proteinase 3
CDK2	cyclin-dependent kinase 2
cGMP	cyclic guanosine monophosphate
CHC	clathrin heavy chain
CO	carbon monoxide
CSNO	S-nitrosocysteine
DHETs	dihydroxyeicosatrienoic acids
Drp1	dynamin-related protein 1
EETs	epoxyeicosatrienoic acids
eIF2Bε	eukaryotic initiation factor 2B epsilon subunit
GPX4	glutathione peroxidase 4
GSSG	oxidized glutathione
IL-1β	interleukin-1β
L-NAME	N^ω^-nitro-l-arginine methyl ester
L-NNA	N^G^-nitro-L-arginine
MAP3K	mitogen-activated protein kinase kinase kinase
MAP4K4	mitogen-activated protein kinase kinase kinase kinase 4
N_2_O_3_	dinitrogen trioxide
NADPH	nicotinamide adenine dinucleotide phosphate
NEMO	NF-κB essential modulator
NLRP3	nod-like receptor protein 3
NSF	*N*-ethylmaleimide-sensitive factor
ODQ	1H-[1,2,4]oxadiazolo [4,3,-a]quinoxalin-1-one
p38MAPK	p38 mitogen-activated protein kinase
PAF	platelet activating factor
PI3K	phosphatidylinositol 3 kinase
PKC	protein kinase C
pRb	retinoblastoma protein
PTEN	phosphatase and TENsin homolog
RIP3	receptor-interacting protein kinase 3
SERCA2	sarcoplasmic/endoplasmic reticulum calcium ATPase 2
SNAP	S-nitro-N-acetylpenicillamine
SNO-MPG	S-nitroso-2-mercaptopropionyl glycine
STAT3	signal transducer and activator of transcription 3
TGFβ	transforming growth factor-β
TLR3	toll-like receptor 3
VE-cadherin	vascular endothelial-cadherin

## References

[1] Stamler J.S., Simon D.I., Osborne J.A., Mullins M.E., Jaraki O., Michel T., Singel D.J., Loscalzo J. (1992). S-nitrosylation of proteins with nitric oxide: Synthesis and characterization of biologically active compounds. Proc. Natl. Acad. Sci. USA.

[2] Phaniendra A., Jestadi D.B., Periyasamy L. (2015). Free radicals: Properties, sources, targets, and their implication in various diseases. Indian J. Clin. Biochem..

[3] Vassileff N., Spiers J.G., Bamford S.E., Lowe R.G.T., Datta K.K., Pigram P.J., Hill A.F. (2024). Microglial activation induces nitric oxide signalling and alters protein S-nitrosylation patterns in extracellular vesicles. J. Extracell. Vesicles.

[4] Wynia-Smith S.L., Smith B.C. (2017). Nitrosothiol formation and S-nitrosation signaling through nitric oxide synthases. Nitric Oxide.

[5] Premont R.T., Reynolds J.D., Zhang R., Stamler J.S. (2020). Role of Nitric Oxide Carried by Hemoglobin in Cardiovascular Physiology: Developments on a Three-Gas Respiratory Cycle. Circ. Res..

[6] Loh K.W.Z., Liang M.C., Soong T.W., Hu Z. (2020). Regulation of cardiovascular calcium channel activity by post-translational modifications or interacting proteins. Pflug. Arch..

[7] Gonzalez M., Clayton S., Wauson E., Christian D., Tran Q.K. (2025). Promotion of nitric oxide production: Mechanisms, strategies, and possibilities. Front. Physiol..

[8] McCarthy O., Moser O., Eckstein M.L., Bain S.C., Pitt J., Bracken R. (2019). Supplementary Nitric Oxide Donors and Exercise as Potential Means to Improve Vascular Health in People with Type 1 Diabetes: Yes to NO?. Nutrients.

[9] Timby N., Domellöf M., Hernell O., Lönnerdal B., Nihlen C., Johanssson I., Weitzberg E. (2020). Effects of age, sex and diet on salivary nitrate and nitrite in infants. Nitric Oxide.

[10] Liao X.H., Xiang Y., Li H., Zheng L., Xu Y., Xi Yu C., Li J.P., Zhang X.Y., Xing W.B., Cao D.S. (2017). VEGF-A Stimulates STAT3 Activity via Nitrosylation of Myocardin to Regulate the Expression of Vascular Smooth Muscle Cell Differentiation Markers. Sci. Rep..

[11] Pinheiro L.C., Oliveira-Paula G.H., Ferreira G.C., Dal-Cin de Paula T., Duarte D.A., Costa-Neto C.M., Tanus-Santos J.E. (2021). Oral nitrite treatment increases S-nitrosylation of vascular protein kinase C and attenuates the responses to angiotensin II. Redox Biol..

[12] Leclerc P.C., Lanctot P.M., Auger-Messier M., Escher E., Leduc R., Guillemette G. (2006). S-nitrosylation of cysteine 289 of the AT1 receptor decreases its binding affinity for angiotensin II. Br. J. Pharmacol..

[13] Hu Z., Zhang B., Lim L.J.Y., Loh W.Z.K., Yu D., Tan B.W.Q., Liang M.C., Huang Z., Leo C.H., Huang H. (2022). S-Nitrosylation-Mediated Reduction of CaV1.2 Surface Expression and Open Probability Underlies Attenuated Vasoconstriction Induced by Nitric Oxide. Hypertension.

[14] Novello M.J., Zhu J., Zhang M., Feng Q., Stathopulos P.B. (2020). Synergistic stabilization by nitrosoglutathione-induced thiol modifications in the stromal interaction molecule-2 luminal domain suppresses basal and store operated calcium entry. Sci. Rep..

[15] Brunner F., Stessel H., Kukovetz W.R. (1995). Novel guanylyl cyclase inhibitor, ODQ reveals role of nitric oxide, but not of cyclic GMP in endothelin-1 secretion. FEBS Lett..

[16] Oliveira-Paula G.H., Batista R.I.M., Stransky S., Tella S.C., Ferreira G.C., Portella R.L., Pinheiro L.C., Damacena-Angelis C., Riascos-Bernal D.F., Sidoli S. (2023). Orally administered sodium nitrite prevents the increased α-1 adrenergic vasoconstriction induced by hypertension and promotes the S-nitrosylation of calcium/calmodulin-dependent protein kinase II. Biochem. Pharmacol..

[17] Ozawa K., Whalen E.J., Nelson C.D., Mu Y., Hess D.T., Lefkowitz R.J., Stamler J.S. (2008). S-nitrosylation of beta-arrestin regulates beta-adrenergic receptor trafficking. Mol. Cell..

[18] Lin L., Xu C., Carraway M.S., Piantadosi C.A., Whorton A.R., Li S. (2018). RhoA inactivation by S-nitrosylation regulates vascular smooth muscle contractive signaling. Nitric Oxide.

[19] Dalle-Donne I., Milzani A., Giustarini D., Di Simplicio P., Colombo R., Rossi R. (2000). S-NO-actin: S-nitrosylation kinetics and the effect on isolated vascular smooth muscle. J. Muscle Res. Cell Motil..

[20] Tang Z.L., Wasserloos K.J., Liu X., Stitt M.S., Reynolds I.J., Pitt B.R., St Croix C.M. (2002). Nitric oxide decreases the sensitivity of pulmonary endothelial cells to LPS-induced apoptosis in a zinc-dependent fashion. Mol. Cell. Biochem..

[21] Chen F., Wang Y., Rafikov R., Haigh S., Zhi W.B., Kumar S., Doulias P.T., Rafikova O., Pillich H., Chakraborty T. (2017). RhoA S-nitrosylation as a regulatory mechanism influencing endothelial barrier function in response to G^+^-bacterial toxins. Biochem. Pharmacol..

[22] Lai Y.C., Pan K.T., Chang G.F., Hsu C.H., Khoo K.H., Hung C.H., Jiang Y.J., Ho F.M., Meng T.C. (2011). Nitrite-mediated S-nitrosylation of caspase-3 prevents hypoxia-induced endothelial barrier dysfunction. Circ. Res..

[23] Palmer L.A., Gaston B., Johns R.A. (2000). Normoxic stabilization of hypoxia-inducible factor-1 expression and activity: Redox-dependent effect of nitrogen oxides. Mol. Pharmacol..

[24] Li J., Zhang Y., Zhang Y., Lü S., Miao Y., Yang J., Huang S., Ma X., Han L., Deng J. (2018). GSNOR modulates hyperhomocysteinemia-induced T cell activation and atherosclerosis by switching Akt S-nitrosylation to phosphorylation. Redox Biol..

[25] Khatib S., Artoul F., Gershko M., Markman G., Vaya J. (2014). The synthesis and analysis of S-nitorsylated paraoxonase 1. Biochem. Biophys. Res. Commun..

[26] Hajouj H., Khattib A., Atrahimovich D., Musa S., Khatib S. (2022). S-Nitrosylation of Paraxonase 1 (PON1) Elevates Its Hydrolytic and Antioxidant Activities. Biomolecules.

[27] Zhao S., Tang X., Miao Z., Chen Y., Cao J., Song T., You D., Zhong Y., Lin Z., Wang D. (2022). Hsp90 S-nitrosylation at Cys521, as a conformational switch, modulates cycling of Hsp90-AHA1-CDC37 chaperone machine to aggravate atherosclerosis. Redox Biol..

[28] Yao Q., Cui Q., Liu J., Xie X., Jiang T., Wang H., Zhao Z., Zhao W., Du X., Lai B. (2023). Free fatty acids stabilize integrin β1via S-nitrosylation to promote monocyte-endothelial adhesion. J. Biol. Chem..

[29] Lai T.S., Hausladen A., Slaughter T.F., Eu J.P., Stamler J.S., Greenberg C.S. (2001). Calcium regulates S-nitrosylation, denitrosylation, and activity of tissue transglutaminase. Biochemistry.

[30] Bekendam R.H., Iyu D., Passam F., Stopa J.D., De Ceunynck K., Muse O., Bendapudi P.K., Garnier C.L., Gopal S., Crescence L. (2018). Protein disulfide isomerase regulation by nitric oxide maintains vascular quiescence and controls thrombus formation. J. Thromb. Haemost..

[31] Zhang G., Li X., Sheng C., Chen X., Chen Y., Zhu D., Gao P. (2016). Macrophages activate iNOS signaling in adventitial fibroblasts and contribute to adventitia fibrosis. Nitric Oxide.

[32] Thenappan T., Ormiston M.L., Ryan J.J., Archer S.L. (2018). Pulmonary arterial hypertension: Pathogenesis and clinical management. BMJ.

[33] Nozik-Grayck E., Whalen E.J., Stamler J.S., McMahon T.J., Chitano P., Piantadosi C.A. (2006). S-nitrosoglutathione inhibits alpha1-adrenergic receptor-mediated vasoconstriction and ligand binding in pulmonary artery. Am. J. Physiol. Lung Cell. Mol. Physiol..

[34] Nozik-Grayck E., McMahon T.J., Huang Y.C., Dieterle C.S., Stamler J.S., Piantadosi C.A. (2002). Pulmonary vasoconstriction by serotonin is inhibited by S-nitrosoglutathione. Am. J. Physiol. Lung Cell. Mol. Physiol..

[35] Ceneviva G.D., Tzeng E., Hoyt D.G., Yee E., Gallagher A., Engelhardt J.F., Kim Y.M., Billiar T.R., Watkins S.A., Pitt B.R. (1998). Nitric oxide inhibits lipopolysaccharide-induced apoptosis in pulmonary artery endothelial cells. Am. J. Physiol..

[36] Sehgal P.B., Lee J.E. (2011). Protein trafficking dysfunctions: Role in the pathogenesis of pulmonary arterial hypertension. Pulm. Circ..

[37] Mukhopadhyay S., Lee J., Sehgal P.B. (2008). Depletion of the ATPase NSF from Golgi membranes with hypo-S-nitrosylation of vasorelevant proteins in endothelial cells exposed to monocrotaline pyrrole. Am. J. physiol. Heart Circ. Physiol..

[38] McMahon T.J., Ahearn G.S., Moya M.P., Gow A.J., Huang Y.C., Luchsinger B.P., Nudelman R., Yan Y., Krichman A.D., Bashore T.M. (2005). A nitric oxide processing defect of red blood cells created by hypoxia: Deficiency of S-nitrosohemoglobin in pulmonary hypertension. Proc. Natl. Acad. Sci. USA.

[39] Grau M., Lauten A., Hoeppener S., Goebel B., Brenig J., Jung C., Bloch W., Suhr F. (2016). Regulation of red blood cell deformability is independent of red blood cell-nitric oxide synthase under hypoxia. Clin. Hemorheol. Microcirc..

[40] Hausladen A., Qian Z., Zhang R., Premont R.T., Stamler J.S. (2022). Optimized S-nitrosohemoglobin Synthesis in Red Blood Cells to Preserve Hypoxic Vasodilation Via βCys93. J. Pharmacol. Exp. Ther..

[41] Fenk S., Melnikova E.V., Anashkina A.A., Poluektov Y.M., Zaripov P.I., Mitkevich V.A., Tkachev Y.V., Kaestner L., Minetti G., Mairbäurl H. (2022). Hemoglobin is an oxygen-dependent glutathione buffer adapting the intracellular reduced glutathione levels to oxygen availability. Redox Biol..

[42] Zhang R., Hausladen A., Qian Z., Liao X., Premont R.T., Stamler J.S. (2022). Hypoxic vasodilatory defect and pulmonary hypertension in mice lacking hemoglobin β-cysteine93 S-nitrosylation. JCI Insight.

[43] Gaston B., May W.J., Sullivan S., Yemen S., Marozkina N.V., Palmer L.A., Bates J.N., Lewis S.J. (2014). Essential role of hemoglobin beta-93-cysteine in posthypoxia facilitation of breathing in conscious mice. J. Appl. Physiol. (1985).

[44] Carver D.J., Gaston B., Deronde K., Palmer L.A. (2007). Akt-mediated activation of HIF-1 in pulmonary vascular endothelial cells by S-nitrosoglutathione. Am. J. Respir. Cell Mol. Biol..

[45] Chen S.C., Huang B., Liu Y.C., Shyu K.G., Lin P.Y., Wang D.L. (2008). Acute hypoxia enhances proteins’ S-nitrosylation in endothelial cells. Biochem. Biophys. Res. Commun..

[46] Ghofrani H.A., Gomberg-Maitland M., Zhao L., Grimminger F. (2025). Mechanisms and treatment of pulmonary arterial hypertension. Nat. Rev. Cardiol..

[47] Yuan D., Chu J., Lin H., Zhu G., Qian J., Yu Y., Yao T., Ping F., Chen F., Liu X. (2023). Mechanism of homocysteine-mediated endothelial injury and its consequences for atherosclerosis. Front. Cardiovasc. Med..

[48] Chen Y., Liu R., Zhang G., Yu Q., Jia M., Zheng C., Wang Y., Xu C., Zhang Y., Liu E. (2015). Hypercysteinemia promotes atherosclerosis by reducing protein S-nitrosylation. Biomed. Pharmacother..

[49] Chen Y., Zhao S., Huang B., Wang Y., Li Y., Waqar A.B., Liu R., Bai L., Fan J., Liu E. (2013). Probucol and cilostazol exert a combinatorial anti-atherogenic effect in cholesterol-fed rabbits. Thromb. Res..

[50] Chakrabarti S., Lekontseva O., Peters A., Davidge S.T. (2010). 17beta-Estradiol induces protein S-nitrosylation in the endothelium. Cardiovasc. Res..

[51] Zhang G., Li C., Zhu N., Chen Y., Yu Q., Liu E., Wang R. (2018). Sex differences in the formation of atherosclerosis lesion in apoE-/-mice and the effect of 17β-estrodiol on protein S-nitrosylation. Biomed. Pharmacother..

[52] Zhu Y., Xian X., Wang Z., Bi Y., Chen Q., Han X., Tang D., Chen R. (2018). Research Progress on the Relationship between Atherosclerosis and Inflammation. Biomolecules.

[53] Boichenko V., Noakes V.M., Reilly-O’Donnell B., Luciani G.B., Emanueli C., Martelli F., Gorelik J. (2025). Circulating Non-Coding RNAs as Indicators of Fibrosis and Heart Failure Severity. Cells.

[54] Bernier T.D., Buckley L.F. (2021). Cardiac Myosin Activation for the Treatment of Systolic Heart Failure. J. Cardiovasc. Pharmacol..

[55] Canton M., Menazza S., Sheeran F.L., Polverino de Laureto P., Di Lisa F., Pepe S. (2011). Oxidation of myofibrillar proteins in human heart failure. J. Am. Coll. Cardiol..

[56] Kirk J.A., Holewinski R.J., Kooij V., Agnetti G., Tunin R.S., Witayavanitkul N., de Tombe P.P., Gao W.D., Van Eyk J., Kass D.A. (2014). Cardiac resynchronization sensitizes the sarcomere to calcium by reactivating GSK-3β. J. Clin. Investig..

[57] Wang S.B., Venkatraman V., Crowgey E.L., Liu T., Fu Z., Holewinski R., Ranek M., Kass D.A., O’Rourke B., Van Eyk J.E. (2018). Protein S-Nitrosylation Controls Glycogen Synthase Kinase 3β Function Independent of Its Phosphorylation State. Circ. Res..

[58] Schiattarella G.G., Altamirano F., Tong D., French K.M., Villalobos E., Kim S.Y., Luo X., Jiang N., May H.I., Wang Z.V. (2019). Nitrosative stress drives heart failure with preserved ejection fraction. Nature.

[59] Guo Y., Wen J., He A., Qu C., Peng Y., Luo S., Wang X. (2023). iNOS contributes to heart failure with preserved ejection fraction through mitochondrial dysfunction and Akt S-nitrosylation. J. Adv. Res..

[60] You H., Gou Q., Dong M., Chang F., Xiu J. (2024). Exploring the role of iNOS in HFpEF-Related myocardial fibrosis: Involvement of PTEN-PI3K/AKT signaling pathway. Biochem. Biophys. Res. Commun..

[61] van der Pol A., van Gilst W.H., Voors A.A., van der Meer P. (2019). Treating oxidative stress in heart failure: Past, present and future. Eur. J. Heart Fail..

[62] Gonzalez D.R., Treuer A.V., Castellanos J., Dulce R.A., Hare J.M. (2010). Impaired S-nitrosylation of the ryanodine receptor caused by xanthine oxidase activity contributes to calcium leak in heart failure. J. Biol. Chem..

[63] Vielma A.Z., Boric M.P., Gonzalez D.R. (2020). Apocynin Treatment Prevents Cardiac Connexin 43 Hemichannels Hyperactivity by Reducing Nitroso-Redox Stress in Mdx Mice. Int. J. Mol. Sci..

[64] Hayashi H., Hess D.T., Zhang R., Sugi K., Gao H., Tan B.L., Bowles D.E., Milano C.A., Jain M.K., Koch W.J. (2018). S-Nitrosylation of β-Arrestins Biases Receptor Signaling and Confers Ligand Independence. Mol. Cell..

[65] Wei J., Duan X., Chen J., Zhang D., Xu J., Zhuang J., Wang S. (2024). Metabolic adaptations in pressure overload hypertrophic heart. Heart Fail. Rev..

[66] Makarewich C.A., Munir A.Z., Schiattarella G.G., Bezprozvannaya S., Raguimova O.N., Cho E.E., Vidal A.H., Robia S.L., Bassel-Duby R., Olson E.N. (2018). The DWORF micropeptide enhances contractility and prevents heart failure in a mouse model of dilated cardiomyopathy. eLife.

[67] Kuhn C., Frank D., Dierck F., Oehl U., Krebs J., Will R., Lehmann L.H., Backs J., Katus H.A., Frey N. (2012). Cardiac remodeling is not modulated by overexpression of muscle LIM protein (MLP). Basic Res. Cardiol..

[68] Tang X., Pan L., Zhao S., Dai F., Chao M., Jiang H., Li X., Lin Z., Huang Z., Meng G. (2020). SNO-MLP (S-Nitrosylation of Muscle LIM Protein) Facilitates Myocardial Hypertrophy Through TLR3 (Toll-Like Receptor 3)-Mediated RIP3 (Receptor-Interacting Protein Kinase 3) and NLRP3 (NOD-Like Receptor Pyrin Domain Containing 3) Inflammasome Activation. Circulation.

[69] Zhao S., Song T.Y., Wang Z.Y., Gao J., Cao J.W., Hu L.L., Huang Z.R., Xie L.P., Ji Y. (2022). S-nitrosylation of Hsp90 promotes cardiac hypertrophy in mice through GSK3β signaling. Acta Pharmacol. Sin..

[70] Li Z., LaPenna K.B., Gehred N.D., Yu X., Tang W.H.W., Doiron J.E., Xia H., Chen J., Driver I.H., Sachse F.B. (2024). Dysregulation of Nitrosylation Dynamics Promotes Nitrosative Stress and Contributes to Cardiometabolic Heart Failure with Preserved Ejection Fraction. bioRxiv.

[71] Tang X., Liu X., Sha X., Zhang Y., Zu Y., Fan Q., Hu L., Sun S., Zhang Z., Chen F. (2025). NEDD4-Mediated GSNOR Degradation Aggravates Cardiac Hypertrophy and Dysfunction. Circ. Res..

[72] Sips P.Y., Irie T., Zou L., Shinozaki S., Sakai M., Shimizu N., Nguyen R., Stamler J.S., Chao W., Kaneki M. (2013). Reduction of cardiomyocyte S-nitrosylation by S-nitrosoglutathione reductase protects against sepsis-induced myocardial depression. Am. J. Physiol. Heart Circ. Physiol..

[73] Tang X., Zhao S., Liu J., Liu X., Sha X., Huang C., Hu L., Sun S., Gao Y., Chen H. (2023). Mitochondrial GSNOR Alleviates Cardiac Dysfunction via ANT1 Denitrosylation. Circ. Res..

[74] Datta Chaudhuri R., Datta R., Rana S., Kar A., Vinh Nguyen Lam P., Mazumder R., Mohanty S., Sarkar S. (2022). Cardiomyocyte-specific regression of nitrosative stress-mediated S-Nitrosylation of IKKγ alleviates pathological cardiac hypertrophy. Cell. Signal..

[75] Tao L., Gao E., Bryan N.S., Qu Y., Liu H.R., Hu A., Christopher T.A., Lopez B.L., Yodoi J., Koch W.J. (2004). Cardioprotective effects of thioredoxin in myocardial ischemia and reperfusion: Role of S-nitrosation [corrected]. Proc. Natl. Acad. Sci. USA.

[76] Chouchani E.T., Methner C., Nadtochiy S.M., Logan A., Pell V.R., Ding S., James A.M., Cochemé H.M., Reinhold J., Lilley K.S. (2013). Cardioprotection by S-nitrosation of a cysteine switch on mitochondrial complex I. Nat. Med..

[77] Bibli S.I., Papapetropoulos A., Iliodromitis E.K., Daiber A., Randriamboavonjy V., Steven S., Brouckaert P., Chatzianastasiou A., Kypreos K.E., Hausenloy D.J. (2019). Nitroglycerine limits infarct size through S-nitrosation of cyclophilin D: A novel mechanism for an old drug. Cardiovasc. Res..

[78] Ding Y., Li Y., Zhang X., He J., Lu D., Fang X., Wang Y., Wang J., Zhang Y., Qiao X. (2017). Soluble epoxide hydrolase activation by S-nitrosation contributes to cardiac ischemia-reperfusion injury. J. Mol. Cell. Cardiol..

[79] Sayed N., Baskaran P., Ma X., van den Akker F., Beuve A. (2007). Desensitization of soluble guanylyl cyclase, the NO receptor, by S-nitrosylation. Proc. Natl. Acad. Sci. USA.

[80] Li X.Y., Zhang H.M., An G.P., Liu M.Y., Han S.F., Jin Q., Song Y., Lin Y.M., Dong B., Wang S.X. (2021). S-Nitrosylation of Akt by organic nitrate delays revascularization and the recovery of cardiac function in mice following myocardial infarction. J. Cell. Mol. Med..

[81] Huang Z.M., Gao E., Fonseca F.V., Hayashi H., Shang X., Hoffman N.E., Chuprun J.K., Tian X., Tilley D.G., Madesh M. (2013). Convergence of G protein-coupled receptor and S-nitrosylation signaling determines the outcome to cardiac ischemic injury. Sci. Signal..

[82] Zhang X., Zhang Y., Miao Q., Shi Z., Hu L., Liu S., Gao J., Zhao S., Chen H., Huang Z. (2021). Inhibition of HSP90 S-nitrosylation alleviates cardiac fibrosis via TGFβ/SMAD3 signalling pathway. Br. J. Pharmacol..

[83] Zhou M., Chen J.Y., Chao M.L., Zhang C., Shi Z.G., Zhou X.C., Xie L.P., Sun S.X., Huang Z.R., Luo S.S. (2022). S-nitrosylation of c-Jun N-terminal kinase mediates pressure overload-induced cardiac dysfunction and fibrosis. Acta Pharmacol. Sin..

[84] Burger D.E., Lu X., Lei M., Xiang F.L., Hammoud L., Jiang M., Wang H., Jones D.L., Sims S.M., Feng Q. (2009). Neuronal nitric oxide synthase protects against myocardial infarction-induced ventricular arrhythmia and mortality in mice. Circulation.

[85] Živković M.L., Zaręba-Kozioł M., Zhukova L., Poznański J., Zhukov I., Wysłouch-Cieszyńska A. (2012). Post-translational S-nitrosylation is an endogenous factor fine tuning the properties of human S100A1 protein. J. Biol. Chem..

[86] Seitz A., Busch M., Kroemer J., Schneider A., Simon S., Jungmann A., Katus H.A., Most P., Ritterhoff J. (2024). S100A1’s single cysteine is an indispensable redox switch for the protection against diastolic calcium waves in cardiomyocytes. Am. J. Physiol. Heart Circ. Physiol..

[87] Power A.S., Asamudo E.U., Worthington L.P.I., Alim C.C., Parackal R.E., Wallace R.S., Ebenebe O.V., Heller Brown J., Kohr M.J., Bers D.M. (2023). Nitric Oxide Modulates Ca^2+^ Leak and Arrhythmias via S-Nitrosylation of CaMKII. Circ. Res..

[88] Gómez R., Caballero R., Barana A., Amorós I., Calvo E., López J.A., Klein H., Vaquero M., Osuna L., Atienza F. (2009). Nitric oxide increases cardiac IK1 by nitrosylation of cysteine 76 of Kir2.1 channels. Circ. Res..

[89] Dallas M.L., Yang Z., Boyle J.P., Boycott H.E., Scragg J.L., Milligan C.J., Elies J., Duke A., Thireau J., Reboul C. (2012). Carbon monoxide induces cardiac arrhythmia via induction of the late Na^+^ current. Am. J. Respir. Crit. Care Med..

[90] Cheng J., Valdivia C.R., Vaidyanathan R., Balijepalli R.C., Ackerman M.J., Makielski J.C. (2013). Caveolin-3 suppresses late sodium current by inhibiting nNOS-dependent S-nitrosylation of SCN5A. J. Mol. Cell. Cardiol..

[91] Yang K.C., Rutledge C.A., Mao M., Bakhshi F.R., Xie A., Liu H., Bonini M.G., Patel H.H., Minshall R.D., Dudley S.C. (2014). Caveolin-1 modulates cardiac gap junction homeostasis and arrhythmogenecity by regulating cSrc tyrosine kinase. Circ. Arrhythm. Electrophysiol..

[92] Bai W., Guo T., Wang H., Li B., Sun Q., Wu W., Zhang J., Zhou J., Luo J., Zhu M. (2024). S-nitrosylation of AMPKγ impairs coronary collateral circulation and disrupts VSMC reprogramming. EMBO Rep..

[93] Okazaki T., Otani H., Shimazu T., Yoshioka K., Fujita M., Katano T., Ito S., Iwasaka T. (2011). Reversal of inducible nitric oxide synthase uncoupling unmasks tolerance to ischemia/reperfusion injury in the diabetic rat heart. J. Mol. Cell. Cardiol..

[94] Xiao C., Xia M.L., Wang J., Zhou X.R., Lou Y.Y., Tang L.H., Zhang F.J., Yang J.T., Qian L.B. (2019). Luteolin Attenuates Cardiac Ischemia/Reperfusion Injury in Diabetic Rats by Modulating Nrf2 Antioxidative Function. Oxid. Med. Cell. Longev..

[95] Chen Y., Li S., Guan B., Yan X., Huang C., Du Y., Yang F., Zhang N., Li Y., Lu J. (2024). MAP4K4 exacerbates cardiac microvascular injury in diabetes by facilitating S-nitrosylation modification of Drp1. Cardiovasc. Diabetol..

[96] Fillmore N., Casin K.M., Sinha P., Sun J., Ma H., Boylston J., Noguchi A., Liu C., Wang N., Zhou G. (2019). A knock-in mutation at cysteine 144 of TRIM72 is cardioprotective and reduces myocardial TRIM72 release. J. Mol. Cell. Cardiol..

[97] Ghanta S.N., Kattamuri L.P.V., Odueke A., Mehta J.L. (2025). Molecular Insights into Ischemia-Reperfusion Injury in Coronary Artery Disease: Mechanisms and Therapeutic Implications: A Comprehensive Review. Antioxidants.

[98] He J., Liu D., Zhao L., Zhou D., Rong J., Zhang L., Xia Z. (2022). Myocardial ischemia/reperfusion injury: Mechanisms of injury and implications for management (Review). Exp. Ther. Med..

[99] Bjørklund G., Zou L., Peana M., Chasapis C.T., Hangan T., Lu J., Maes M. (2022). The Role of the Thioredoxin System in Brain Diseases. Antioxidants.

[100] Al-Kandari N., Fadel F., Al-Saleh F., Khashab F., Al-Maghrebi M. (2019). The Thioredoxin System is Regulated by the ASK-1/JNK/p38/Survivin Pathway During Germ Cell Apoptosis. Molecules.

[101] Obsilova V., Honzejkova K., Obsil T. (2021). Structural Insights Support Targeting ASK1 Kinase for Therapeutic Interventions. Int. J. Mol. Sci..

[102] Leary P.J., Rajasekaran S., Morrison R.R., Tuomanen E.I., Chin T.K., Hofmann P.A. (2008). A cardioprotective role for platelet-activating factor through NOS-dependent S-nitrosylation. Am. J. Physiol. Heart Circ. Physiol..

[103] Sun J., Picht E., Ginsburg K.S., Bers D.M., Steenbergen C., Murphy E. (2006). Hypercontractile female hearts exhibit increased S-nitrosylation of the L-type Ca^2+^ channel alpha1 subunit and reduced ischemia/reperfusion injury. Circ. Res..

[104] Shi J., Yu Y., Yuan H., Li Y., Xue Y. (2025). Mitochondrial dysfunction in AMI: Mechanisms and therapeutic perspectives. J. Transl. Med..

[105] Nadtochiy S.M., Burwell L.S., Ingraham C.A., Spencer C.M., Friedman A.E., Pinkert C.A., Brookes P.S. (2009). In vivo cardioprotection by S-nitroso-2-mercaptopropionyl glycine. J. Mol. Cell. Cardiol..

[106] Kohr M.J., Sun J., Aponte A., Wang G., Gucek M., Murphy E., Steenbergen C. (2011). Simultaneous measurement of protein oxidation and S-nitrosylation during preconditioning and ischemia/reperfusion injury with resin-assisted capture. Circ. Res..

[107] Huang H., Ruan Y., Li C., Zheng H., Tang Y., Chen Y., He F., Liu Y., Wu G., Li Z. (2025). Hypoxia Microenvironment Preconditioning Attenuated Myocardial Ischemia-Reperfusion Injury via Stc1-Mediating Cardiomyocyte Self-Protection and Neutrophil Polarization. Adv. Sci..

[108] Sayed N., Kim D.D., Fioramonti X., Iwahashi T., Durán W.N., Beuve A. (2008). Nitroglycerin-induced S-nitrosylation and desensitization of soluble guanylyl cyclase contribute to nitrate tolerance. Circ. Res..

[109] Oppermann M., Suvorava T., Freudenberger T., Dao V.T., Fischer J.W., Weber M., Kojda G. (2011). Regulation of vascular guanylyl cyclase by endothelial nitric oxide-dependent posttranslational modification. Basic Res. Cardiol..

[110] Talman V., Ruskoaho H. (2016). Cardiac fibrosis in myocardial infarction-from repair and remodeling to regeneration. Cell Tissue Res..

[111] Treuer A.V., Gonzalez D.R. (2014). NOS1AP modulates intracellular Ca^2+^ in cardiac myocytes and is up-regulated in dystrophic cardiomyopathy. Int. J. Physiol. Pathophysiol. Pharmacol..

[112] Puebla M., Muñoz M.F., Lillo M.A., Contreras J.E., Figueroa X.F. (2024). Control of astrocytic Ca^2+^ signaling by nitric oxide-dependent S-nitrosylation of Ca^2+^ homeostasis modulator 1 channels. Biol. Res..

[113] Rohde D., Ritterhoff J., Voelkers M., Katus H.A., Parker T.G., Most P. (2010). S100A1: A multifaceted therapeutic target in cardiovascular disease. J. Cardiovasc. Transl. Res..

[114] Dhamoon A.S., Jalife J. (2005). The inward rectifier current (IK1) controls cardiac excitability and is involved in arrhythmogenesis. Heart Rhythm..

[115] Gaborit N., Le Bouter S., Szuts V., Varro A., Escande D., Nattel S., Demolombe S. (2007). Regional and tissue specific transcript signatures of ion channel genes in the non-diseased human heart. J. Physiol..

[116] Ueda K., Valdivia C., Medeiros-Domingo A., Tester D.J., Vatta M., Farrugia G., Ackerman M.J., Makielski J.C. (2008). Syntrophin mutation associated with long QT syndrome through activation of the nNOS-SCN5A macromolecular complex. Proc. Natl. Acad. Sci. USA.

[117] Kleindienst A., Battault S., Belaidi E., Tanguy S., Rosselin M., Boulghobra D., Meyer G., Gayrard S., Walther G., Geny B. (2016). Exercise does not activate the β3 adrenergic receptor-eNOS pathway, but reduces inducible NOS expression to protect the heart of obese diabetic mice. Basic Res. Cardiol..

[118] Chen Q.M., Maltagliati A.J. (2018). Nrf2 at the heart of oxidative stress and cardiac protection. Physiol. Genom..

[119] Cinelli M.A., Do H.T., Miley G.P., Silverman R.B. (2020). Inducible nitric oxide synthase: Regulation, structure, and inhibition. Med. Res. Rev..

[120] Shi X., O’Connor M., Qiu H. (2024). Valosin-containing protein acts as a target and mediator of S-nitrosylation in the heart through distinct mechanisms. Redox Biol..

[121] Chen Y.J., Lu C.T., Su M.G., Huang K.Y., Ching W.C., Yang H.H., Liao Y.C., Chen Y.J., Lee T.Y. (2015). dbSNO 2.0: A resource for exploring structural environment, functional and disease association and regulatory network of protein S-nitrosylation. Nucleic. Acids Res..

[122] Aboalroub A.A., Al Azzam K.M. (2024). Protein S-Nitrosylation: A Chemical Modification with Ubiquitous Biological Activities. Protein J..

[123] Shi X., Qiu H. (2020). Post-Translational S-Nitrosylation of Proteins in Regulating Cardiac Oxidative Stress. Antioxidants.

[124] Trepel J., Mollapour M., Giaccone G., Neckers L. (2010). Targeting the dynamic HSP90 complex in cancer. Nat. Rev. Cancer.

[125] Kar A., Gupta S., Matilal A., Sarkar S. (2025). Tissue engineering with targeted delivery of nanotized S-nitrosyl mutant of NEMO ameliorates myocardial infarction. Nanomedicine.

[126] Choi M., Choi W., Hwang P.T.J., Oh Y., Jun T., Ryu D.Y., Kim N.K., Jang E.H., Shin Y.R., Youn Y.N. (2025). Engineered silk fibroin bio-hybrid artificial graft with releasing biological gas for enhanced circulatory stability and surgical performance. Int. J. Biol. Macromol..

